# Photoactuating Artificial Muscles of Motor Amphiphiles as an Extracellular Matrix Mimetic Scaffold for Mesenchymal Stem Cells

**DOI:** 10.1021/jacs.1c12318

**Published:** 2022-02-16

**Authors:** Shaoyu Chen, Liangliang Yang, Franco King-Chi Leung, Takashi Kajitani, Marc C. A. Stuart, Takanori Fukushima, Patrick van Rijn, Ben L. Feringa

**Affiliations:** † Center for System Chemistry, Stratingh Institute for Chemistry, University of Groningen, AG Groningen 9747, The Netherlands; ‡ Department of Biomedical Engineering, University Medical Center Groningen, University of Groningen, AV Groningen 9713, The Netherlands; § Laboratory for Chemistry and Life Science, Institute of Innovative Research, Tokyo Institute of Technology, 4259 Nagatsuta, Midori-ku, Yokohama 226-8503, Japan; ∥ Key Laboratory for Advanced Materials and Joint International Research Laboratory of Precision Chemistry and Molecular Engineering, Feringa Nobel Prize Scientist Joint Research Center, Frontiers Science Center for Materiobiology and Dynamic Chemistry, Institute of Fine Chemicals, School of Chemistry and Molecular Engineering, East China University of Science and Technology, Shanghai 200237, China

## Abstract

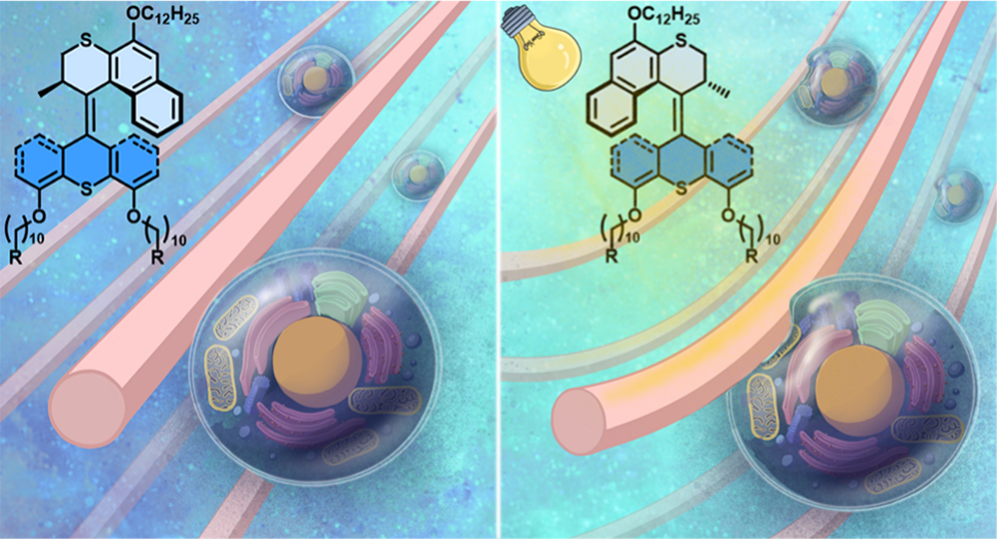

Mimicking the native extracellular matrix (ECM) as a cell culture scaffold has long attracted scientists from the perspective of supramolecular chemistry for potential application in regenerative medicine. However, the development of the next-generation synthetic materials that mimic key aspects of ECM, with hierarchically oriented supramolecular structures, which are simultaneously highly dynamic and responsive to external stimuli, remains a major challenge. Herein, we present supramolecular assemblies formed by motor amphiphiles (**MAs**), which mimic the structural features of the hydrogel nature of the ECM and additionally show intrinsic dynamic behavior that allow amplifying molecular motions to macroscopic muscle-like actuating functions induced by light. The supramolecular assembly (named artificial muscle) provides an attractive approach for developing responsive ECM mimetic scaffolds for human bone marrow-derived mesenchymal stem cells (**hBM-MSCs**). Detailed investigations on the photoisomerization by nuclear magnetic resonance and UV–vis absorption spectroscopy, assembled structures by electron microscopy, the photoactuation process, structural order by X-ray diffraction, and cytotoxicity are presented. Artificial muscles of **MAs** provide fast photoactuation in water based on the hierarchically anisotropic supramolecular structures and show no cytotoxicity. Particularly important, artificial muscles of **MAs** with adhered **hBM-MSCs** still can be actuated by external light stimulation, showing their ability to convert light energy into mechanical signals in biocompatible systems. As a proof-of-concept demonstration, these results provide the potential for building photoactuating ECM mimetic scaffolds by artificial muscle-like supramolecular assemblies based on **MAs** and offer opportunities for signal transduction in future biohybrid systems of cells and **MAs**.

## Introduction

Hierarchical supramolecular polymeric structures are commonly found in living systems, for example, the extracellular matrix (ECM), cell membranes, and the cytoskeleton. They serve vital roles in key biological functions, allowing for cell growth, division, and movement.^1−5^ The ECM is a three-dimensional (3D) supramolecular network mainly consisting of fibrous proteins and proteoglycans, present within all tissues and organs, which provides not only important physical support for the surrounding cells but also crucial biochemical and biomechanical signals in a dynamic and spatiotemporal manner for tissue morphogenesis, differentiation, and homeostasis.^6^ Taking inspiration from the essential roles of natural ECM in governing cell functions in living systems, numerous synthetic polymers have been developed for ECM mimics, ranging from 1D to 3D soft materials, providing promising potential for tissue engineering and regenerative medicine, such as stem cell-based therapies.^7−11^ Due to the dynamic and adaptive nature of native ECM, supramolecular polymers, including thermoplastic elastomers^7^ on the basis of polyurethanes, bisurea, and ureidopyrimidinone motifs as well as hydrogels, are attractive candidates, which have been applied in vitro or in vivo for tissue regeneration.^7,9,12−15^ Among the above biomaterials, hydrogels are able to absorb up to 99% of water, allowing for the encapsulation and diffusion of cells under physiological conditions, which provide a 3D environment more closely resembling the native ECM situation.^7−9^ A special class of supramolecular hydrogels formed by peptide amphiphile assemblies, reported by the Stupp group, is among the most remarkable materials showing unique advantages of injectability, biocompatibility, and biodegradability.^16−18^ These peptide amphiphiles are generally composed of alternating hydrophobic alkyl chains and hydrophilic amino acid residues, which spontaneously form well-organized assemblies ranging from nanoscale to macroscopic 3D isotropic or anisotropic hydrogels.^16^ Isotropic hydrogels, developed from peptide amphiphiles combined with epitopes^19−22^ or growth factors^23,24^ or mimicked bioactive sequences,^25−29^ have been used to mimic the ECM, showing possible clinical applications in bone therapy;^27,30^ brain,^31^ kidney,^32^ and central nervous system injury;^33^ cartilage regeneration;^34^ and angiogenesis.^35,36^ Compared to isotropic hydrogels, anisotropic hydrogels with hierarchically high-oriented structures are considered a particularly attractive class of ECM mimetic scaffold for regenerative medicine.^37,38^ They are important for tissues with unidirectionally aligned structures, such as muscle fibers, the spinal cord, bones, and parts of the brain.^37^ Particularly, by gently shearing the annealed solution of peptide amphiphiles into salt-containing media, highly oriented noodle-like strings with an arbitrary length can be obtained which perform as templates to induce the alignment of growth direction of cells,^39^ showing challenging applications in regenerative medicine, for example, for blood vessels,^40^ neural tissues,^41−43^ bones,^44^ cavernous nerves,^45^ and muscle tissues.^46^ Except for the hierarchically anisotropic supramolecular structure, the ideal ECM mimetic scaffold for regenerative medicine also should be highly dynamic and responsive, allowing for adaptiveness and spatiotemporal feedback control on cell functions as a result of trigger signals or changes in the environment.^7,17^ Only very few studies reported responsive ECM mimetic scaffolds by using enzymatic cleavage or photocleavage to remove bioactive sequences, typically being irreversible in nature.^47,48^ In the approach presented here, different advantageous material attributes are included, however, to the best of our knowledge prior to this study, and no single system has been found to be able to simultaneously satisfy the following multiple requirements, that is, to provide biocompatible supramolecular materials with hierarchically highly oriented structures, high dynamics, as well as response to external stimuli in a non-invasive manner and amplification of mechanical effects across the length scale from molecular motions to macroscopic movements, which is likely a significant step toward the development of next-generation dynamic ECM mimetic scaffolds for tissue engineering and regenerative medicine.^7,17,18^ It should be emphasized that the synthetic systems mimic only certain aspects of ECM, that is, in our design, biocompatibility with living cells and hierarchical organization in water and while integrating this with our light-driven motor function to allow non-invasive dynamic functions.

Recently, we have demonstrated the first example of a hierarchically organized anisotropic supramolecular system comprising a motor amphiphile (**MA**) in aqueous media, allowing for the development of reversible photoactuating artificial muscles in water and in air.^49^ These artificial muscles were prepared by self-assembly involving the addition of **MA** aggregates (composed of 95% water) to a CaCl_2_ solution using a shear flow method for alignment.^49−51^ The unique hierarchically anisotropic supramolecular structures enabled the amplification of molecular motions across length scales. Note that our systems, in contrast to natural muscles, are triggered by light as a non-invasive stimulus to induce macroscopic photoactuation, allowing for light energy conversion to mechanical motions. Because these **MA** artificial muscles meet multiple requirements of potential next-generation ECM mimetic scaffolds, we envisioned that they might be biocompatible and be applied in systems controlling the fate of cells.

**Figure 1:**
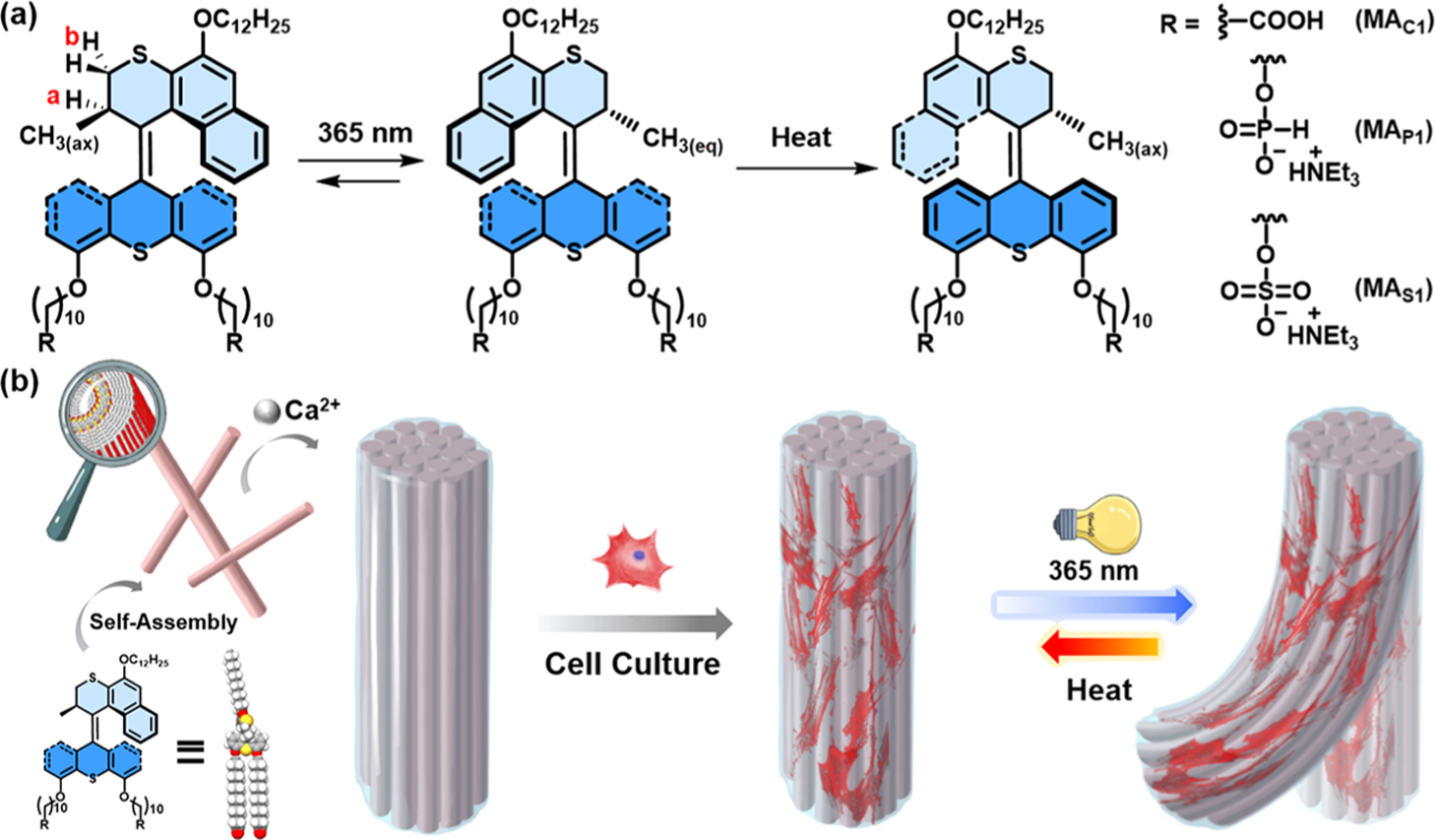
Schematic illustration of (a) reversible photoisomerization and thermal helix inversion of molecular **MAs** and (b) hierarchically anisotropic supramolecular assembly structures of photoactuating artificial muscles of **MAs** and the application of **MA** artificial muscles as ECM mimetic scaffolds for mesenchymal stem cells.

Here, we present **MAs**, which are based on the second-generation of the molecular motor core,^52−56^ combined with a hydrophobic alkyl chain and hydrophilic chains with various end groups, that is, analogues of charged groups in the native ECM including carboxylate groups (**MA**_**C1**_), phosphite groups (**MA**_**P1**_), and sulfate groups (**MA**_**S1**_), as shown in Figure 1. After investigating the effect of the functionalities on the molecular isomerization process and supramolecular assembly in water, the **MAs** are used to prepare photoactuating artificial muscles with highly oriented supramolecular structures. For the first time, these structurally well-oriented artificial muscles are combined with human bone marrow-derived mesenchymal stem cells (**hBM-MSCs**) to determine the cytotoxicity and mechanical motion in the presence of cells. As a proof-of-concept demonstration, we explored the possibility of photoactuating artificial muscles of **MAs** as ECM mimetic scaffolds for mesenchymal stem cells. The prospects of transducing mechanical signals to control cell functions in the next stage might lead to potential applications such as in vitro pathology models for studying complicated cell signaling response environments and to ultimately provide stem cell-based therapies that address challenging medical problems in the future, such as nerve tissue regeneration after spinal cord injuries.

## Results and Discussion

### Molecular Design and Synthesis

**Scheme 1:**
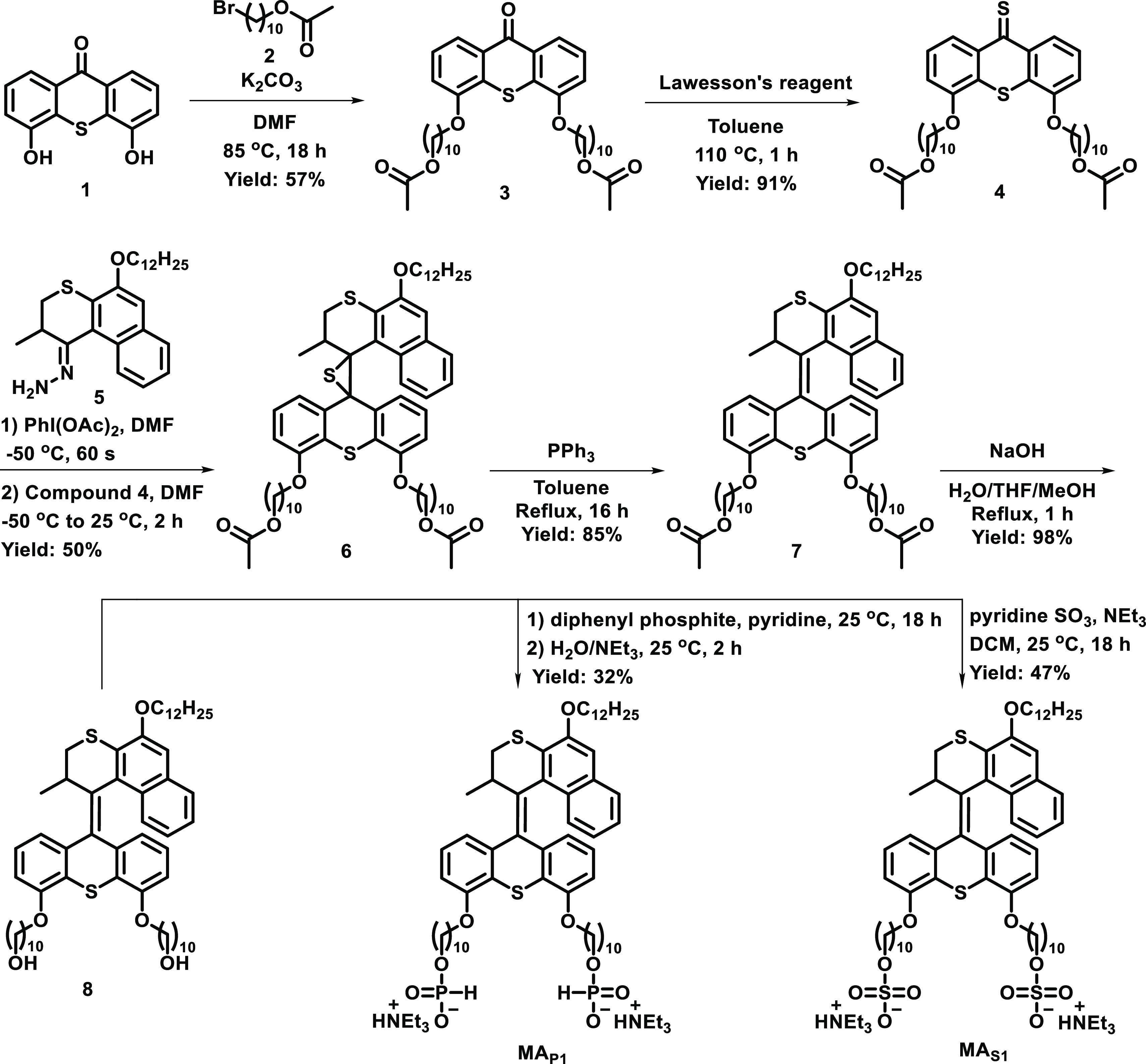
Synthesis of **MAs**

Our earlier **MAs**,^49,50^ for forming artificial muscles, were designed with a dodecyl chain attached to the upper half of a second-generation molecular motor core and two carboxylate end groups connected with two alkyl linkers to the lower half of the motor core. By considering the common phosphorylated and sulfated groups in the native ECM, these functional groups play important roles in the mineralization and providing cation binding sites.^28,57^ In this context, the end groups of **MA** were extended from carboxylate groups (**MA**_**C1**_) to the analogues of naturally existing charged groups (Figure 1), that is, phosphite groups (**MA**_**P1**_) and sulfate groups (**MA**_**S1**_), which not only allow identifying the end-group effects on molecular isomerization processes and assembled structures but also provide biocompatible groups, allowing for potential applications in developing photoactuating artificial muscles as photoresponsive ECM mimetic scaffolds for mesenchymal stem cells. The synthetic pathways for the **MAs** are summarized in Scheme 1.

Stator and rotor units, that is, compounds **1** and **5**, were prepared by our reported procedures.^49,58^ Compound **3** was obtained from thioxanthone **1** and alkyl bromide **2** by a Williamson ether formation reaction in the presence of K_2_CO_3_ in DMF and subsequently converted into thioketone **4** with Lawesson’s reagent in toluene. Hydrazone **5** was oxidized in situ with (diacetoxyiodo)benzene in DMF to provide the corresponding diazo-compound, followed by the addition of freshly prepared thioketone **4**, providing the corresponding episulfide **6**. Desulfurization with triphenylphosphine in toluene gave overcrowded alkene **7**, which was hydrolyzed to provide diol **8**. The new **MAs** were obtained via two different synthetic methods: functionalization of **8** with diphenyl phosphite yielded **MA**_**P1**_,^59^ while **MA**_**S1**_ was obtained by a sulfate ester formation of **8** with a sulfur trioxide pyridine complex and subsequent hydrolysis.^60^ The structures of all new compounds were established by ^1^H, ^13^C nuclear magnetic resonance (NMR) and high-resolution ESI mass spectrometry, and detailed synthetic procedures and data are provided in the Supporting Information (SI: pages S3–S6, Figures S9–S20).

### Photoisomerization and Thermal Helix Inversion of MAs

**Figure 2:**
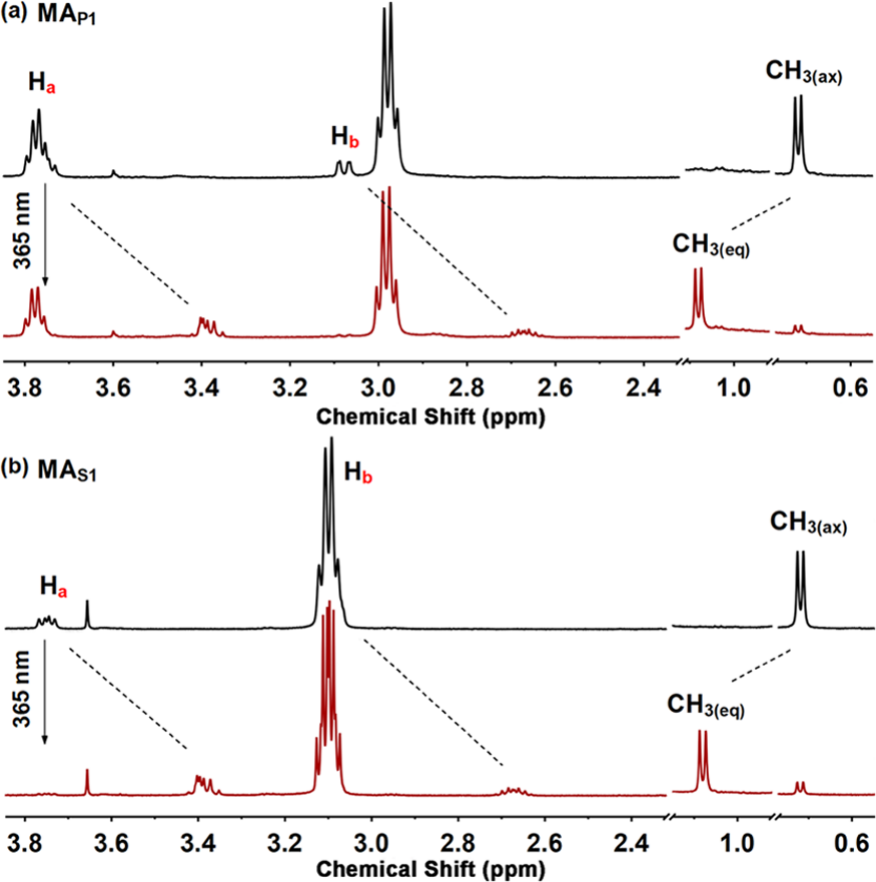
Selected regions of ^1^H NMR spectra (CD_2_Cl_2_, 25 °C, 500 MHz) of (a) **MA**_**P1**_ and (b) **MA**_**S1**_ in a stable state (black) and a mixture (red) containing 85% metastable isomers at PSS after irradiation (for the proton assignment, see Figure 1, for full spectra, see Figure S2).

**Figure 3:**
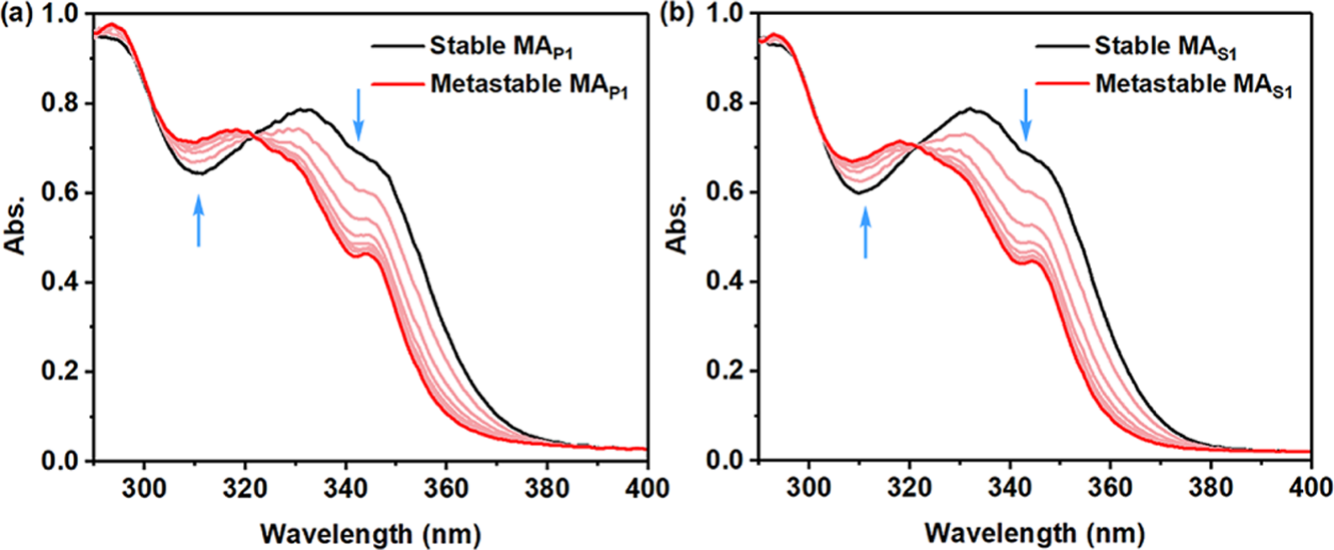
UV–vis absorption spectra of (a) **MA**_**P1**_ and (b) **MA**_**S1**_ in CH_3_CN solutions (6.5 × 10^–2^ mM) before 365 nm light irradiation (black), upon irradiation from 1 to 3 min (pink), and after irradiation to PSS (red).

The photochemical and thermal isomerization steps of **MA**_**P1**_ and **MA**_**S1**_ were examined by ^1^H NMR and UV–vis spectroscopy. Essentially identical proton signal shifts of H_a_, H_b_, and the methyl group adjacent to the stereogenic center are observed in the ^1^H NMR spectra of CD_2_Cl_2_ solutions of **MA**_**P1**_ and **MA**_**S1**_, and upon prolonged irradiation with 365 nm light, photostationary states (PSS) with metastable/stable isomer ratios of 85:15 are obtained for both **MA**_**P1**_ and **MA**_**S1**_ (Figures 2, S2 and Table S1). In the UV–vis absorption spectra of CH_3_CN solutions of **MA**_**P1**_ and **MA**_**S1**_, an increase in the absorption around 310 nm with a concomitant decrease of the absorption band from 330 to 370 nm is observed upon irradiating with 365 nm light, which is essentially identical to that observed in **MA**_**C1**_,^49^ indicating the isomerization from a stable configuration to a metastable configuration (Figure 3). Additionally, a clear isosbestic point at 327 nm over the course of irradiation indicates that a selective photoisomerization process occurs (Figure 3). The transformation from the metastable isomer into the stable isomer can be induced by heating. The thermal helix inversion processes of **MA**_**P1**_ and **MA**_**S1**_ in CH_3_CN solutions were studied by means of Eyring analysis (Figure S3 and Table S2). The activation parameters and half-life of **MA**_**P1**_ and **MA**_**S1**_ are presented in Table S2. For example, the Gibbs free energy of activation (Δ^‡^*G*) of **MA**_**P1**_ was 102.5 kJ mol^–1^, which corresponded to a half-life (*t*_1/2_) of 27.9 h at 25 °C. In UV–vis absorption spectra of MAs in water (Figure S4), similar spectra changes are observed, which are consistent with the spectra performed in CH_3_CN (Figure 3). The results of the photoisomerization and thermal helix inversion of **MA**_**P1**_ and **MA**_**S1**_ are comparable to **MA**_**C1**_,^49^ indicating that selective molecular isomerization processes between stable and metastable isomers are observed typical for the second generation motors by light and subsequent heat stimuli. This provides a solid basis for the further investigation of the amplification of molecular motion to macroscopic photoactuation.

### Supramolecular Assembly and Photoactuation

**Figure 4:**
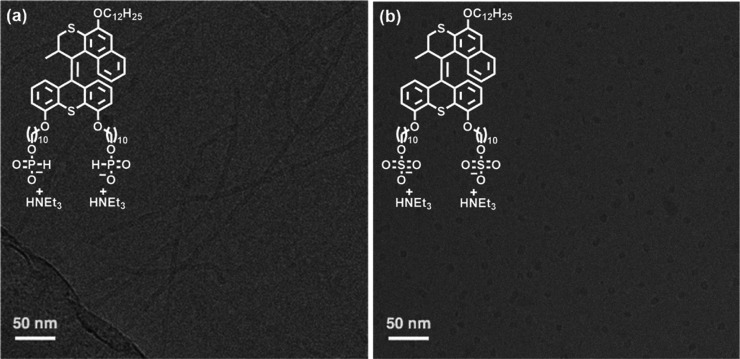
Cryo-TEM images of aqueous solutions of (a) **MA**_**P1**_ and (b) **MA**_**S1**_ (concentration: 3.9 mM, above the CAC).

Aqueous solutions of **MA**_**P1**_ or **MA**_**S1**_ were prepared by direct dissolution into double deionized water or tris-buffer (pH 7.4). The corresponding aqueous solutions of **MAs** were heated at 80 °C for 30 min and subsequently cooled down to room temperature to afford colorless transparent solutions. The critical aggregation concentration (CAC) of MAs was measured by using a Nile Red fluorescence assay (NRFA), which probes the internal hydrophobicity of the assemblies.^50,61,62^ Significantly smaller blue shifts of the emission wavelength of Nile Red are observed when aqueous solutions of **MAs** are diluted below 0.01 mM, and the CACs of **MA**_**P1**_ and **MA**_**S**1_ are determined to be 0.76 and 1.51 μM, respectively (Figure S5). Freshly prepared solutions of **MAs** at a concentration of 3.9 mM, above the CAC, were used to analyze the self-assembled structures by using cryogenic transmission electron microscopy (cryo-TEM) to capture their solution-state morphologies. **MA**_**P1**_ assembles into worm-like micelles from hundreds of nanometers to micrometers in length and ∼5–6 nm in diameter (Figure 4a), which is reminiscent of the assemblies of **MA**_**C1**_.^49−51^ Compared to the worm-like micelles in **MA**_**C1**_ and **MA**_**P1**_, significantly different micellar-type assemblies (∼10–20 nm in diameter) are observed in **MA**_**S1**_ (Figure 4b), indicating that the sulfate end groups (**MA**_**S1**_) results in a lower packing parameter than the amphiphilic motors with the carboxylate (**MA**_**C1**_) and phosphite (**MA**_**P1**_) end groups (e.g., micelles: *P* ≤ 1/3, worm-like micelles: 1/3 < *P* ≤ 1/2).^63−65^ Considering the assemblies of **MA**_**C1**_,^49−51^ the results imply a possible formation of photoactuating artificial muscles by **MA**_**P1**_.

**Figure 5:**
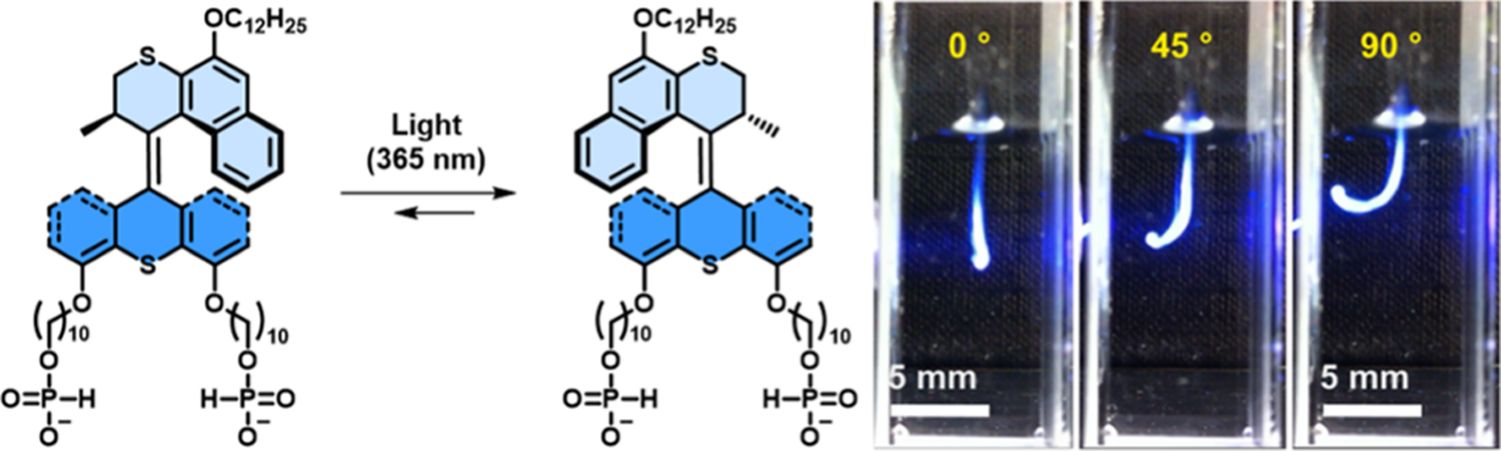
Photoisomerization step of **MA**_**P1**_ and photoactuation of an **MA**_**P1**_ artificial muscle after irradiation with 365 nm light for 60 s.

**Figure 6:**
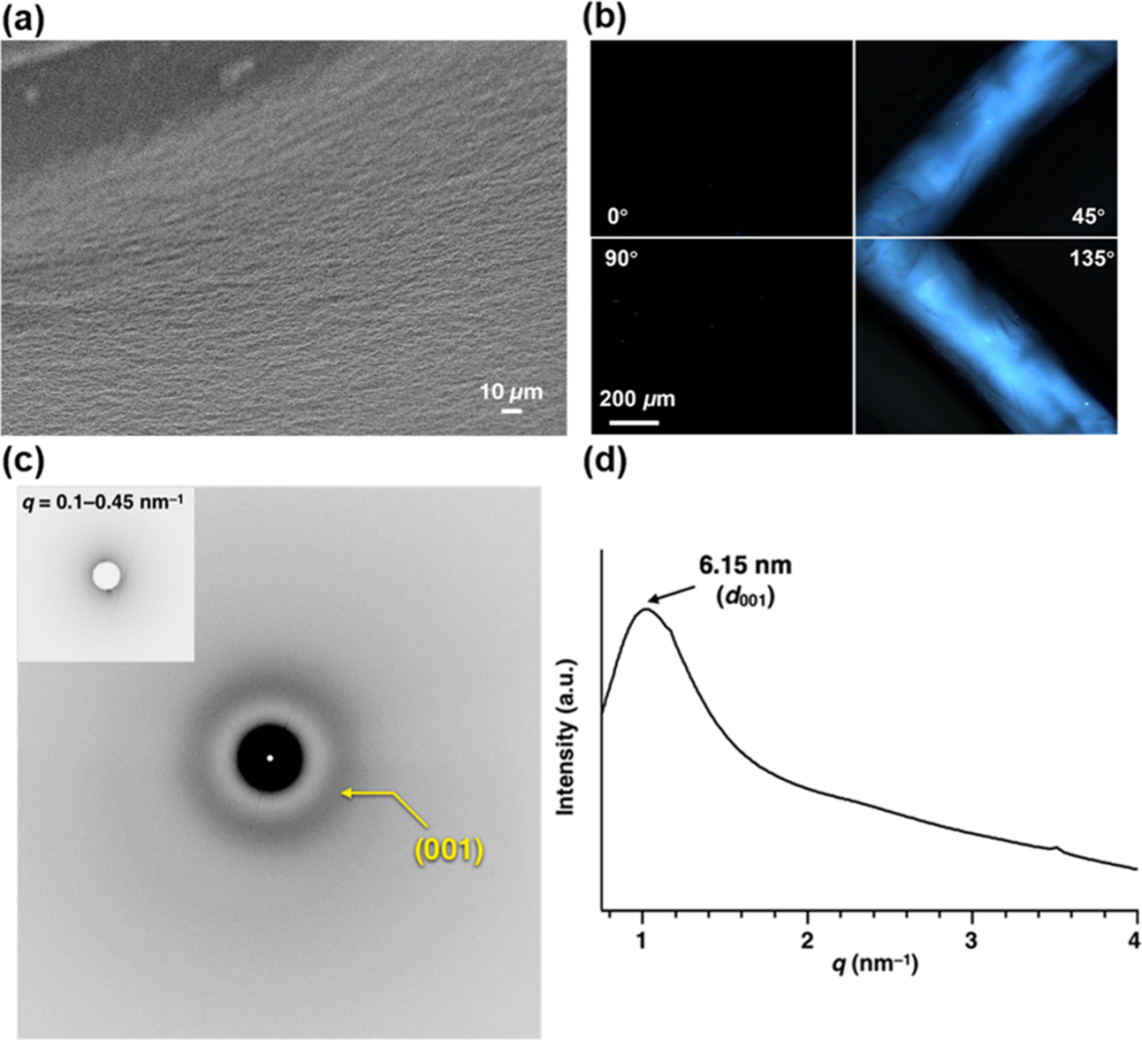
(a) SEM and (b) POM images of a noodle-like artificial muscle of **MA**_**P1**_ under crossed polarizers. The POM images of **MA**_**P1**_ artificial muscle were tilted at 0, 45, 90, and 135° relative to the transmission axis of the analyzer. A scale bar applied for all panels. For images taken at other angles, see Figure S6. (c) 2D SAXS image of the **MA**_**P1**_ artificial muscle (inset: enlarged 2D image for *q* = 0.1–0.45 nm^–1^ at 25 °C). (d) 1D SAXS patterns of the **MA**_**P1**_ artificial muscle of 2D SAXS images in (c), showing the diffraction pattern in the direction perpendicular to the long axis of the artificial muscle.

The **MA** artificial muscles were formed according to our reported procedure.^49−51^ Typically, an aqueous solution of **MAs** (55 mM) was manually drawn into an aqueous solution of CaCl_2_ (150 mM) from a pipette by a shear flow method, allowing for the formation of a noodle-like artificial muscle with an arbitrary length. As expected, noodle-like artificial muscles were obtained from a **MA**_**P1**_ solution, while a direct dissolution of **MA**_**S1**_ into the media was observed. Therefore, only **MA**_**P1**_ was further investigated regarding photoactuation and structural features, and both **MA**_**C1**_ and **MA**_**P1**_ were investigated in cell culture studies. An artificial muscle of **MA**_**P1**_ is prepared in a cuvette containing CaCl_2_ solution, which bends toward the light source from an initial position of 0° to a flexion angle of 90°, upon 365 nm irradiation for 60 s, with an actuating speed of 1.5 ± 0.2°/s (Figure 5). Compared to the actuating speed of the **MA**_**C1**_ artificial muscle (7.9 ± 0.4°/s) under identical conditions, different actuation speeds can be obtained by changing the end groups, which could be attributed to the effect of end groups on the molecular packing, the calcium-ion binding and the degree of alignment of the assembly structures.^49−51^ Next, the structural properties of the **MA**_**P1**_ artificial muscle, including the morphology and structural orientation, were characterized by using scanning electron microscopy (SEM), polarized optical microscopy (POM), and through-view small-angle X-ray scattering (SAXS) technique, and the results are shown in Figure 6.

The SEM image of an **MA**_**P1**_ artificial muscle in Figure 6a shows arrays of unidirectionally aligned bundles of worm-like micelles, and the POM images of a freshly prepared **MA**_**P1**_ artificial muscle present uniform birefringence in the direction of the long axis of the muscle (Figure 6b). The structural parameters and orientational orders, that is, degree of alignment, of the **MA**_**P1**_ worm-like micelles in the macroscopic noodle-like **MA**_**P1**_ artificial muscle were obtained by through-view SAXS measurements. In the 2D SAXS image of the **MA**_**P1**_ artificial muscle, prepared on a sapphire substrate at 25 °C (Figure 6c), a pair of spot-like scatterings is observed in a smaller-angle region (*q* = 0.1–0.45 nm^–1^) (Figure 6c, inset), which is due to scattering from the unidirectionally aligned bundles of worm-like micelles. The diffraction arcs with a *d*-spacing of 6.15 nm (Figure 6d) are attributed to the diffraction from the (001) plane of a lamellar structure, which is constructed by the unidirectionally aligned worm-like micelles of **MA**_**P1**_ with ionic interaction between Ca^2+^ and phosphite of **MA**_**P1**_ as interfibrillar interaction. The layer spacing of the lamellar structure (*c* = 6.15 nm) of the **MA**_**P1**_ artificial muscle is longer than that observed in **MA**_**C1**_ (*c* = 5.48 nm),^50^ possibly due to a loose packing between **MA**_**P1**_ worm-like micelles and Ca^2+^. The angular dependency of the peak intensity of the diffraction from the (001) plane, converted from the through-view 2D SAXS image of the **MA**_**P1**_ artificial muscle, shows intensity maxima at 0 and 180° (Figure 6c). The peak intensity of the diffraction from the (001) plane was quantified by full-width half-maximum (fwhm) to obtain an ∼100° azimuthal angle, in which a smaller azimuthal angle represented a higher degree of unidirectional alignment. Given that the **MA**_**C1**_ artificial muscle showed an ∼65° azimuthal angle,^50^ the results indicated that a lower degree of unidirectional alignment was present in the **MA**_**P1**_ artificial muscle. The results demonstrated that likely due to the effects of end-groups in the molecular MAs on molecular packing and structural orientation in supramolecular assembly, artificial muscles of **MA**s with different photoactuating speeds can be obtained.

### Cell Culture and Post-photoactuation

**Figure 7:**
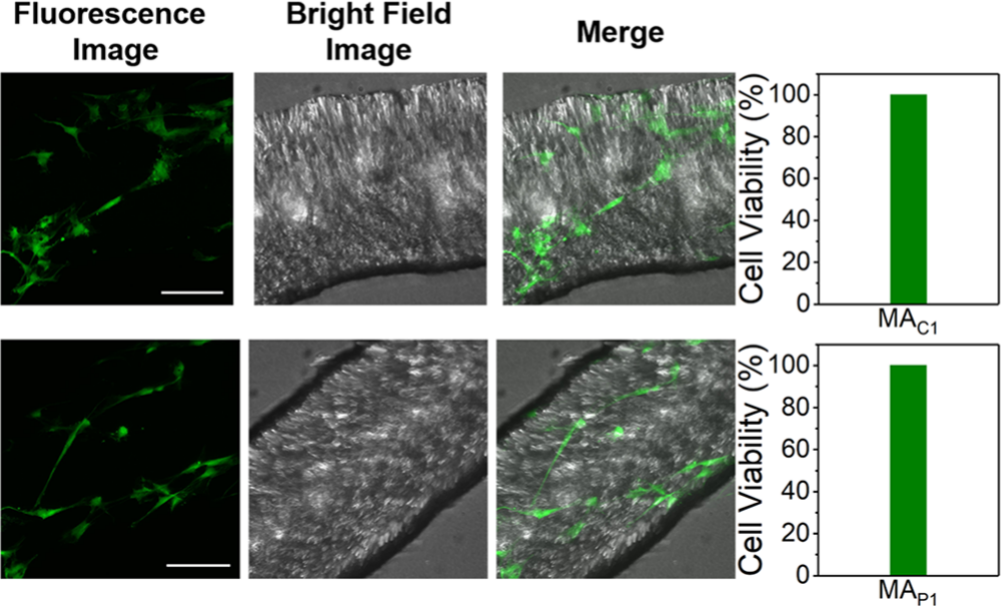
Cytotoxicity of artificial muscles of **MA**_**C1**_ (top) and **MA**_**P1**_ (bottom) for **hBM-MSCs** after 24 h of incubation, determined by a live/dead staining assay. The images were taken by confocal laser scanning microscopy. Scale bar: 100 μm, applied for all panels. The bar graphs (right) show the percentages of live cells in the fluorescent images (for full images, see Figure S7).

To explore the potential of **MA** artificial muscles as ECM mimetic scaffolds for cell culture, **hBM-MSCs** were selected because of their typical advantages toward differentiation possibilities and potential future clinical translation.^33^ Following an identical preparation protocol for **MA** artificial muscles, freshly prepared artificial muscles of **MA**_**C1**_ and **MA**_**P1**_, as ECM mimetic scaffolds, were placed in the 24-well plates containing growth medium (0.5 mL), followed by evenly seeding **hBM-MSCs** into the well plates with a density of 20,000 cells/well. **hBM-MSCs** were expected to randomly attach to the surface of MA artificial muscles (for details procedure of the cell culture, see Supporting Information, SI page S8). After incubation for 24 h, the cytotoxicity was analyzed by the live/dead assay by using calcein-AM and ethidium homodimer-1 in PBS to stain **hBM-MSCs**. After staining, the **MA** artificial muscles were transferred from the original 24-well plates to a glass plate or Petri dish, which avoided significant background fluorescence from **hBM-MSCs** attached on the surface of the 24-well plates for the subsequent cell observation under confocal laser scanning microscopy and fluorescence microscopy. Live cells show green fluorescence due to the uptake of calcein-AM via intracellular activity, while dead cells show red fluorescence because of the entry of ethidium homodimer-1 through the damaged cell membranes and its subsequent binding to nucleic acids. The predominantly observed green fluorescence and in the absence of red fluorescence strongly suggested an almost 100% cell viability of **hBM-MSCs** cultured in the artificial muscles of **MA**_**C1**_ and **MA**_**P1**_ (Figures 7 and S7). Furthermore, it is shown that the live cells are attached to the surface of the artificial muscle (Figures 7 and S7). Using a direct contact method between **hBM-MSCs** and **MA** artificial muscles, **MA** artificial muscles, as ECM mimetic scaffolds, showed no cytotoxicity, indicating a good in vitro biocompatibility of **MA** artificial muscles.^66^

**Figure 8:**
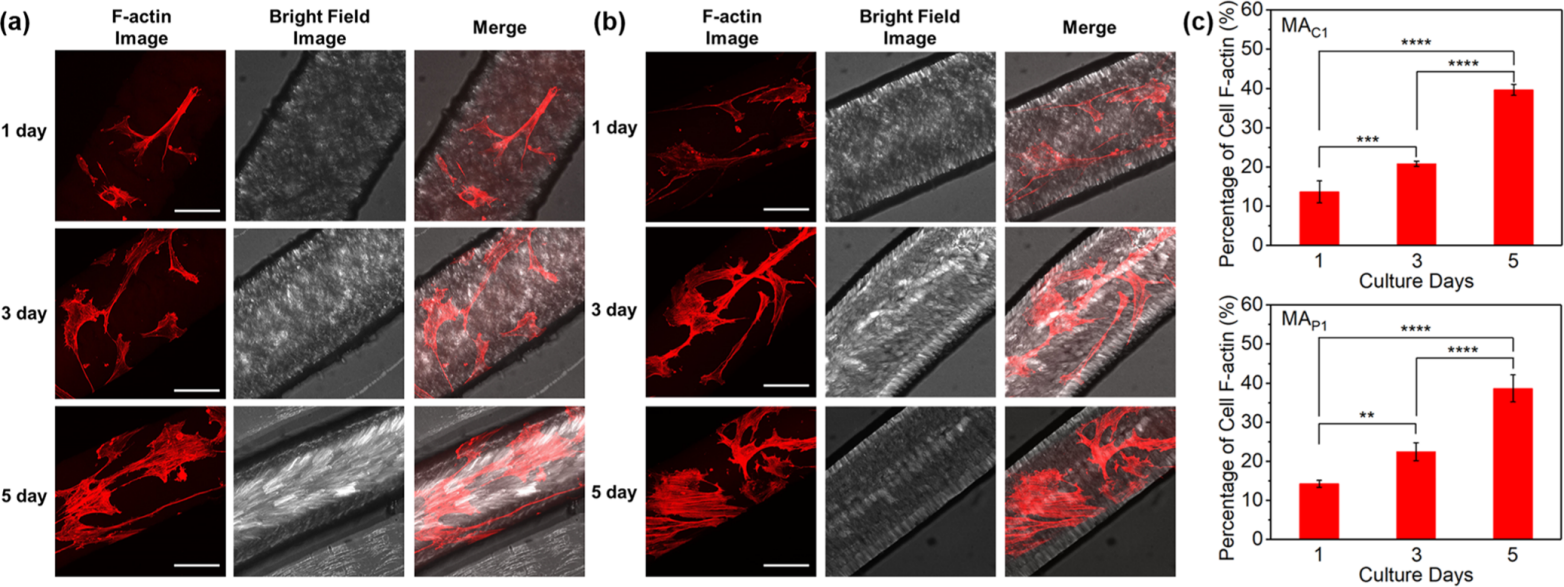
F-actin of **hBM-MSCs** on the surface of artificial muscles of (a) **MA**_**C1**_ and (b) **MA**_**P1**_. The images were taken by confocal laser scanning microscopy. Scale bar: 100 μm, applied for all panels. **hBM-MSCs** are stained using phalloidin for F-actin (red). The bar graphs (c) show the changes of average percentages of cell F-actin coverage on the surface of artificial muscles of **MA**_**C1**_ (top) and **MA**_**P1**_ (bottom) over various culture days, indicating a possibility of cell proliferation. The percentage of cell F-actin coverage was detected by dividing the area of F-actin by the total surface area of **MA** artificial muscles. A value of 100% indicates that the artificial muscle is completely covered. Data are shown as mean (±) standard deviation (SD), and **P < 0.01, ***P < 0.001, ****P < 0.0001.

To provide a deeper insight into the condition of **hBM-MSCs** cultured on the surface of the **MA** artificial muscles, the **hBM-MSCs** were seeded into 24-well plates (with a density of 2500 cells/well) containing a growth medium and freshly prepared **MA** artificial muscles, incubating for 1, 3, and 5 d, and then 4′,6-diamidino-2-phenylindole (DAPI) and tetramethylrhodamine isothiocyanate(TRITC)–phalloidin were used to stain cell nuclei and F-actins of **hBM-MSCs**, respectively. Subsequently, the corresponding **MA** artificial muscles with adhered **hBM-MSCs** were transferred from the original 24-well plates to a glass plate and were observed using confocal laser scanning microscopy (for details protocol, see Supporting Information, SI page S8). Generally, a small and round cell shape is typically an indication of cells entering apoptosis,^67,68^ whereas a well-spread cell shape of **hBM-MSCs** is commonly quantified as being in a viable state.^69−71^ As depicted in Figure 8a,b and Supporting Information, Movie 1, cells spread with well-defined actin stress fibers on the surfaces of **MA** artificial muscles, indicating that **hBM-MSCs** maintain highly viable over prolonged incubation time. Due to overlapping fluorescence between the strong background of **MA** artificial muscles and the cell nuclei stained by DAPI, the change of cell F-actin area, instead of the number of cell nuclei, was used to indicate the cell proliferation. The cell F-actin area significantly increases upon prolonged incubation time from 1 to 5 d, suggesting a possibility of cell proliferation of **hBM-MSCs** on the surfaces of **MA** artificial muscles (Figure 8a,b). To further provide a quantitative analysis of the change of F-actin area, the percentage of F-actin coverage was calculated by dividing the area of F-actin by the total surface area of **MA** artificial muscles (Figure 8c). The areas were measured by the software of ImageJ.^69^ Both in the **MA**_**C1**_ and **MA**_**P1**_ artificial muscles, the F-actin coverages show significant increase from ∼15% (1 d culture) to ∼22% (3 d), and to ∼40% (5 d), which not only suggests a highly viable state of **hBM-MSCs** but also indicates a possibility of good cell proliferation (Figure 8c).

**Figure 9:**
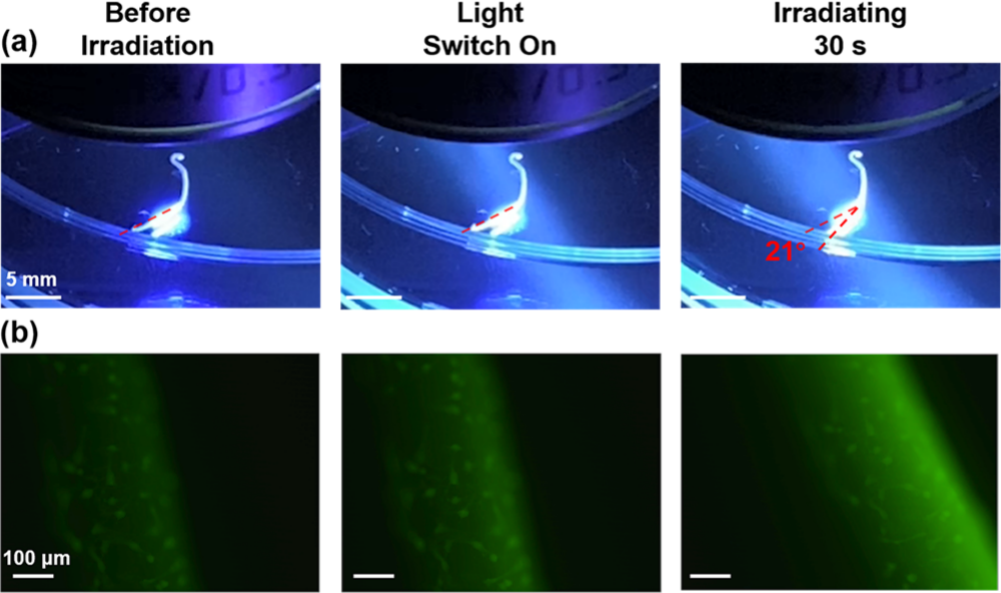
Snapshots showing (a) macroscopic photoactuation (scale bar: 5.0 mm) of an **MA**_**C1**_ artificial muscle with adhered **hBM-MSCs** on the surface and (b) in situ observation of photoactuating movement accompanied with cell visualization under fluorescence microscopy upon 365 nm light irradiation for 30 s (scale bar: 100 μm), demonstrating the maintaining of photoactuating function of **MA** artificial muscles after their application as ECM mimetic scaffolds for mesenchymal stem cells.

In addition to the excellent viability of **hBM-MSCs** cultured in the **MA** artificial muscles, it should be emphasized that **MA** artificial muscles with adhered **hBM-MSCs** maintain their photoactuation function (Movies 2, 3, Figures 9 and S8). During the macroscopic photoactuation of **MA** artificial muscles with adhered **hBM-MSCs** on the surface, the in situ photoactuating process was monitored under fluorescence microscopy and simultaneously by digital movies (Figure 9 and Movie 2, Figure S8 and Movie 3). The method allows showing the photoactuating movement accompanied with a cell visualization (live cells stained by using calcein-AM). The **MA**_**C1**_ artificial muscle with adhered **hBM-MSCs** bends toward the light source from an initial position of 0° to a flexion angle of 21° upon 365 nm photoirradiation for 30 s (Figure 9). Upon prolonged photoirradiation to 60 s, an increased flexion angle to 40° is obtained (Movie 2). Compared to **MA**_**C1**_, due to a loose packing between worm-like micelles of **MA**_**P1**_ and Ca^2+^ as well as a lower degree of unidirectional alignment (Figure 6), a slower photoactuating speed of **MA**_**P1**_ artificial muscles with adhered **hBM-MSCs** is observed, that is, the **MA**_**P1**_ artificial muscle bends from an initial position of 0° to a flexion angle of 21° upon 365 nm photoirradiation for 200 s (Movie 3, Figure S8), again indicating that the photoactuating speeds of **MA** artificial muscles with adhered **hBM-MSCs** can be tuned by the end-groups. The results demonstrated that **MA** artificial muscles, as responsive ECM mimetic scaffolds for **hBM-MSCs**, showed no cytotoxicity. Particularly important, **MA** artificial muscles with adhered **hBM-MSCs** maintain their photoactuation function with tunable actuation speed, demonstrating the capability of photoenergy conversion into mechanical actuation in the presence of **hBM-MSCs**. We demonstrated that a multifunctional synthetic–biological system in water, comprising artificial muscles of **MAs** and mesenchymal stem cells, is capable of converting light energy into mechanical actuation from the molecular level to macroscopic dimensions. As a proof-of-concept study, these results provide attractive applications of photoactuating artificial muscles of **MAs** as ECM mimetic scaffolds for **hBM-MSCs**, suggesting opportunities for the transduction of a mechanical actuation signal to control cell functions in the future.

## Conclusions

Molecular **MAs** with variously charged end groups, that is, carboxylate groups (**MA**_**C1**_), phosphite groups (**MA**_**P1**_), and sulfate groups (**MA**_**S1**_), were designed to form artificial muscles. Both artificial muscles of **MA**_**C1**_ and **MA**_**P1**_ allow fast photoactuation due to the amplification of molecular motor motion along length scales. This is based on the distinct isomerization processes of the motor units and the hierarchically anisotropic assembly structures in water, as confirmed by UV–vis absorption and NMR spectroscopy, electron microscopy, and X-ray diffraction. Taking artificial muscles of **MA**_**C1**_ and **MA**_**P1**_ as ECM mimetic scaffolds, they show no cytotoxicity, and particularly important, **MA** artificial muscles with adhered **hBM-MSCs** still can be actuated by photoirradiation. This study shows the feasibility of developing the next generation of ECM mimetic scaffolds by using artificial muscles of **MAs** with highly oriented supramolecular structures and the ability to convert light energy into mechanical actuation in biological systems. Following this proof of concept, the possibility of using **MA** artificial muscles as responsive scaffolds for mesenchymal stem cells, using visible light-driven artificial muscles is envisioned. This will enable to systematically investigate cell properties and explore the transduction of mechanical actuation signal to control the differentiation of mesenchymal stem cells, which is a part of our ongoing program with future prospects toward responsive materials for regenerative medicine.

## Supporting Information Available

Synthesis, ^1^H and ^13^C NMR spectra, parameters of thermal helix inversion, UV–vis absorption spectra, Nile Red fluorescence assay, optical and polarized optical microscopy images, fluorescence microscopy images, and photoactuating process images (PDF)
Three-dimensional visualization of **MA** artificial muscles with adhered **hBM-MSCs** by using confocal laser scanning microscopy (MP4)
Post-photoactuation of the **MA**_**C1**_ artificial muscle with adhered **hBM-MSCs** (MP4)
Post-photoactuation of the **MA**_**P1**_ artificial muscle with adhered **hBM-MSCs** (MP4)
