# Supplementary material for: Photoactuating Artificial Muscles of Motor Amphiphiles as an Extracellular Matrix Mimetic Scaffold for Mesenchymal Stem Cells
Source: J Am Chem Soc. 2022 Feb 16;144(8):3543–53. doi: 10.1021/jacs.1c12318 (PMC8895399; doi:10.1021/jacs.1c12318)
Supplement: Supplementary file 1 — ja1c12318_si_001.pdf [file ja1c12318_si_001.pdf]

Supporting Information

Photoactuating Artificial Muscles of Motor Amphiphiles as an Extracellular Matrix Mimetic Scaffold for Mesenchymal Stem Cells

Shaoyu Chen[†,§], Liangliang Yang[#], Franco King-Chi Leung[†,$,*], Takashi Kajitani[‡], Marc C. A. Stuart[†], Takanori Fukushima[‡], Patrick van Rijn[#,*], Ben L. Feringa[†,§,*]

[†]Center for System Chemistry, Stratingh Institute for Chemistry, University of Groningen, 9747 AG Groningen, The Netherlands.
[#]University of Groningen, Department of Biomedical Engineering, University Medical Center Groningen, 9713 AV Groningen, The Netherlands.
[‡]Laboratory for Chemistry and Life Science, Institute of Innovative Research, Tokyo Institute of Technology, 4259 Nagatsuta, Midori-ku, Yokohama 226-8503, Japan.
[§]Key Laboratory for Advanced Materials and Joint International Research Laboratory of Precision Chemistry and Molecular Engineering, Feringa Nobel Prize Scientist Joint Research Center, Frontiers Science Center for Materiobiology and Dynamic Chemistry, Institute of Fine Chemicals, School of Chemistry and Molecular Engineering, East China University of Science and Technology, Shanghai 200237, China.

[*]To whom correspondence should be addressed.
E-mail: kingchifranco.leung@polyu.edu.hk; p.van.rijn@umcg.nl; b.l.feringa@rug.nl

Present Addresses

[$]F.K.C.L.: State Key Laboratory of Chemical Biology and Drug Discovery, Department of Applied Biology and Chemical Technology, The Hong Kong Polytechnic University, Hong Kong (China).

Table of Contents

Materials and Methods
1. Materials
2. General
3. Synthesis
4. Nile Red Fluorescence Assay
5. Cryogenic Transmission Electron Microscopy
6. Standardized Preparation of Artificial Muscle of Motor Amphiphile in Water
7. Synchrotron Radiation X-ray Diffraction Analysis
8. Scanning Electron Microscopy and Polarized Optical Microscopy Analysis
9. Cell Culture
10. Cytotoxicity and Cell Viability Analysis
11. Post-Photoactuation Experiment of Artificial Muscles with Adhered **hBM-MSCs**
12. Description of Supplementary Movies

^{1}H NMR Spectra of Photoisomerization of **MAs** (Figure S2, Table S1)
Kinetic Studies of Thermal Inversion Step of **MA$_{P1}$** and **MA$_{S1}$** in Acetonitrile (Figure S3, Table S2)
UV-vis Absorption Spectra of Photoisomerization of **MAs** in Water (Figure S4)
Nile Red Fluorescence Assay for Determining Critical Aggregation Concentration (Figure S5)
Structural Analysis of **MA$_{P1}$** Artificial Muscle by Optical and Polarized Optical Microscopy (Figure S6)
Fluorescence Microscopy Images for Cytotoxicity (Figure S7)
Post-Photoactuation of **MA$_{P1}$** Artificial Muscle with adhered **hBM-MSCs** (Figure S8)
Analytical Data (Figure S9-S20)
References

Materials and Methods

1. Materials. All commercial reagents and solvents were purchased from Aldrich, TCI, Acros, Fluka, or Merck, which were used as received unless otherwise stated. All reactions were performed under an inert atmosphere (Nitrogen). Analytical TLC was performed with Merck silica gel 60 F254 plates and visualization was accomplished by UV light. Flash chromatography was carried out using Merck silica gel 60 (230-400 mesh ASTM). Solvents for spectroscopic studies were of spectrophotometric grade (UVASOL Merck). Compounds **4**, *i.e.*, 5-dihyderoxy-9*H*-thioxanthen-9-one (**1**)[1] and hydrazone (**5**)[2] were synthesized according to our previously reported procedures.

2. General. NMR spectra were recorded at 25 °C on Varian AMX400 (^{1}H: 400 MHz, ^{13}C: 100 MHz), Varian Unity Plus (^{1}H: 500 MHz, ^{13}C: 125 MHz), or Bruker Avance 600 (^{1}H: 600 MHz, ^{13}C: 150 MHz) NMR spectrometers. The deuterated solvents (CD_2Cl_2 and $CDCl_3$) were treated with Na_2CO_3 and molecular sieves (4Å), followed by degassing with argon prior to use. Chemical shifts (δ) are expressed relative to the resonances of the residual non-deuterated solvent for ^{1}H [$CDCl_3$: ^{1}H(δ) = 7.26 ppm, CD_2Cl_2: ^{1}H(δ) = 5.32 ppm] and ^{13}C [$CDCl_3$: ^{13}C(δ) = 77.16 ppm]. Absolute values of the coupling constants are given in Hertz (Hz), regardless of their sign. Multiplicities are abbreviated as singlet (s), doublet (d), doublet of doublets (dd), triplet (t), multiplet (m), and broad (br). High-resolution mass spectrometry (HRMS) was performed on an LTQ Orbitrap XL spectrometer with electrospray ionization (ESI) technique. UV-vis measurements were performed on a Hewlett-Packard HP 8543 Diode Array UV-vis Spectrophotometer in a 1 cm path length quartz cuvette. The UV-vis samples were irradiated by using a LED light from Thorlabs (M365FP1, 0.2 A) at 20 °C, positioned at a distance of 1.0 cm from the samples. Photoactuation experiments were carried out with a LED light from Thorlabs (M365FP1, 0.7 A).

3. Synthesis.

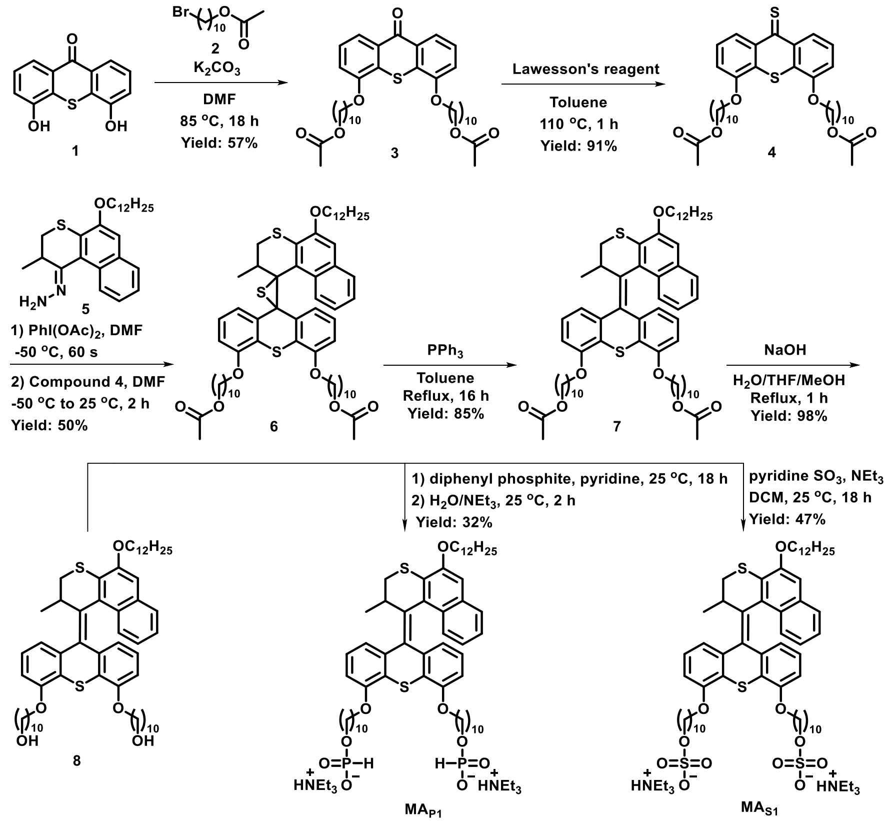

Compound 3:

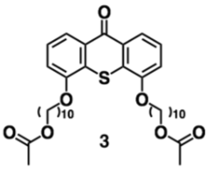

To a suspension of compound **1** (180 mg, 0.74 mmol) and K_2CO_3 (407 mg, 2.95 mmol) in 20 mL DMF, alkyl bromide **2** (516 mg, 1.85 mmol) was added at 25 °C, whereupon the mixture was stirred at 85 °C for 18 h. After cooling down to room temperature, the reaction mixture was poured into water (20 mL) and subsequently washed with water (40 mL) and ethyl acetate (20 mL). The combined organic layers were washed with brine and dried over Na_2SO_4, followed by removing the solvent under reduced pressure. The residue was subjected to column chromatography on SiO_2 (ethyl acetate/ pentane; v/v = 1/5, R_f = 0.5) to allow for the isolation of compound **3** (268 mg, 0.42 mmol, 57% yield) as a pale yellow powder.

^{1}H NMR (400 MHz, $CDCl_3$) δ (ppm) 8.18 (d, J = 8.0 Hz, 2H), 7.35 (dd, J = 8.0, 8.0 Hz, 2H), 7.06 (d, J = 8.0 Hz, 2H), 4.12 (t, J = 6.4 Hz, 4H), 4.00 (t, J = 6.8 Hz, 4H), 2.00 (s, 6H), 1.91 – 1.84 (m, 4H), 1.60 – 1.49 (m, 8H), 1.40 – 1.28 (m, 20H).

^{13}C NMR (100 MHz, $CDCl_3$) δ (ppm) 180.3, 171.1, 154.4, 130.0, 128.4, 125.8, 121.2, 113.1, 69.4, 64.6, 29.5, 29.5, 29.3, 29.3, 29.0, 28.6, 26.0, 26.0, 21.0.

HRMS (ESI): calcd. for $C_{37}H_{52}O_7S$ [M+Na] 663.3326, found 663.3302.

Compound 6:

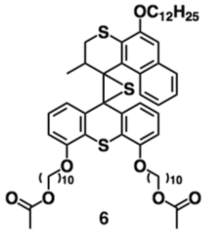

A mixture of compound **3** (267 mg, 0.42 mmol) and Lawesson's reagent (680 mg, 1.7 mmol) in toluene (10 mL) was stirred at 110 °C for 1 h. After solvent removal under reduced pressure, the residue was purified by column chromatography on SiO_2 (ethyl acetate/ pentane; v/v = 1/5, R_f = 0.5) to obtain thioketone (252 mg, 0.38 mmol, 91% yield), *i.e.*, compound **4**, as a green solid. Subsequently, a DMF solution (2 mL) of (diacetoxyiodo)benzene (123.7 mg, 0.384 mmol) was added dropwise into a THF solution (3 mL) of compound **5** (164 mg, 0.384 mmol) at –50 °C. After complete addition, the obtained pale pink solution was stirred at –50 °C for 60 s, followed by the addition of a THF (2 mL) solution of compound **4**. The reaction mixture was stirred at 25 °C for 2 h. Subsequently, the solvent was removed by using rotary evaporation under reduced pressure and the residue was subjected to column chromatography on SiO_2 (ethyl acetate/pentane; v/v = 1/9, R_f = 0.5) to allow for the isolation of episulfide (191 mg, 0.181 mmol, 50% yield), *i.e.*, compound **6,** as a colorless oil.

^{1}H NMR (400 MHz, $CDCl_3$) δ (ppm) 8.61 (d, J = 8.4 Hz, 1H), 7.67 (d, J = 8.0 Hz, 1H), 7.50 (d, J = 8.0 Hz, 1H), 7.37 – 7.27 (m, 2H), 7.20 (dd, J = 8.0, 8.0 Hz, 1H), 6.86 (d, J = 8.0 Hz, 1H), 6.70 (s, 1H), 6.31 (m, 2H), 6.12 (dd, J = 8.0, 8.0 Hz, 1H), 4.23 – 4.12 (m, 1H), 4.04 (m, 5H), 3.95 – 3.77 (m, 4H), 2.65 – 2.52 (m, 2H), 2.11 (m, 1H), 2.04 (s, 6H), 1.99 – 1.69 (m, 6H), 1.68 – 1.23 (m, 46H), 1.18 (d, J = 6.0 Hz, 3H), 0.89 (t, J = 6.0 Hz, 3H).

^{13}C NMR (100 MHz, $CDCl_3$) δ (ppm) 193.8, 171.3, 154.5, 153.6, 152.8, 135.0, 132.6, 131.5, 131.2, 130.7, 129.7, 127.0, 126.2, 124.9, 124.5, 124.3, 124.3, 124.1, 123.4, 122.7, 121.9, 110.1, 109.9, 106.1, 69.7, 69.5, 69.5, 65.5, 64.8, 61.9, 40.8, 35.7, 32.1, 29.9, 29.8, 29.8, 29.7, 29.7, 29.7, 29.6, 29.6, 29.5, 29.5, 29.5, 29.4, 29.4, 29.1, 28.8, 28.7, 26.2, 26.2, 26.1, 26.1, 26.1, 22.8, 21.1, 14.3.

HRMS (ESI): calcd. for $C_{63}H_{88}O_7S_3$ [M+H] 1053.5765, found 1053.5748.

Compound 7:

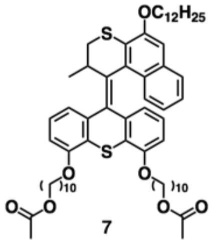

7

A mixture of episulfide (compound **6**, 191 mg, 0.181 mmol) and triphenylphosphine (95 mg, 0.36 mmol) in toluene (10 mL) was stirred at reflux for 16 h. After cooling down to room temperature, the solvent was removed under reduced pressure and the residue was subjected to column chromatography on SiO_2 (ethyl acetate/pentane; v/v = 1/9, R_f = 0.4) to allow for the isolation of compound **7** (158 mg, 0.155 mmol, 85% yield) as a colorless oil.

^{1}H NMR (400 MHz, $CDCl_3$) δ (ppm) 7.47 (m, 2H), 7.28 (dd, J = 7.6, 7.6 Hz, 1H), 7.18 (d, J = 7.6 Hz, 1H), 7.05 (dd, J = 7.6, 7.6 Hz, 1H), 6.94 (s, 1H), 6.87 – 6.82 (m, 2H), 6.36 (dd, J = 7.6, 7.6 Hz, 1H), 6.28 (d, J = 8.0 Hz, 1H), 6.08 (d, J = 7.6 Hz, 1H), 4.21 – 4.13 (m, 3H), 4.08 – 4.05 (m, 6H), 4.00 – 3.86 (m, 2H), 3.75 (dd, J = 11.6, 6.8 Hz, 1H), 3.10 (dd, J = 11.6, 2.8 Hz, 1H), 2.05 (s, 6H), 1.99 – 1.90 (m, 4H), 1.85 – 1.78 (m, 2H), 1.67 – 1.27 (m, 46H), 0.90 (t, J = 6.0 Hz, 3H), 0.77 (d, J = 6.8 Hz, 3H).

^{13}C NMR (100 MHz, $CDCl_3$) δ (ppm) 193.9, 171.3, 155.7, 154.7,153.0, 138.9, 136.5, 135.8, 132.2, 132.0, 131.7, 127.9, 126.5, 126.3, 126.1, 125.5, 125.2, 124.6, 124.6, 123.9, 123.0, 121.8, 119.7, 109.4, 109.4, 105.1, 69.2, 69.1, 68.8, 64.8, 36.8, 32.1, 30.6, 29.8, 29.8, 29.8, 29.7, 29.7, 29.7, 29.7, 29.6, 29.6, 29.5, 29.5, 29.4, 29.3, 29.3, 28.8, 26.3, 26.2, 26.1, 26.1, 22.8, 21.1, 18.5, 14.3.

HRMS (ESI): calcd. for $C_{63}H_{88}O_7S_2$ [M+H] 1021.6044, found 1021.6017.

Compound 8:

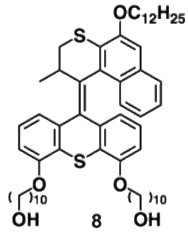

8

To a THF/methanol solution (v/v = 1/1, 6 mL) of compound **7** (158 mg, 0.155 mmol), an aqueous NaOH solution (4 M, 0.34 mL, 1.4 mmol) was added and the mixture was stirred at 90 °C for 1 h. The reaction mixture was allowed to cool down to room temperature. The solvent was removed under reduced pressure and then the residue was washed successively with water (50 mL) and ethyl acetate (20 mL). The combined organic layers were washed with brine and dried over Na_2SO_4. The solvent was removed under reduced pressure and the residue was purified by column chromatography on SiO_2 (methanol/dichloromethane; v/v = 1/19, R_f = 0.5) to obtain compound **8** (142 mg, 0.151 mmol, 98% yield) as a colorless oil.

^{1}H NMR (400 MHz, $CDCl_3$) δ (ppm) 7.45 (m, 2H), 7.28 (dd, J = 8.0, 8.0 Hz, 1H), 7.18 (d, J = 7.6 Hz, 1H), 7.05 (dd, J = 7.2, 7.2 Hz, 1H), 6.94 (s, 1H), 6.87 – 6.82 (m, 2H), 6.36 (dd, J = 8.0, 8.0 Hz, 1H), 6.27 (d, J = 8.0 Hz, 1H), 6.08 (d, J = 7.6 Hz, 1H), 4.21 – 4.12 (m, 3H), 4.10 – 4.04 (m, 2H), 3.99 – 3.86 (m, 2H), 3.75 (dd, J = 11.6, 6.8 Hz, 1H), 3.64 (t, J = 6.8 Hz, 4H), 3.10 (dd, J = 11.2, 2.4 Hz, 1H), 1.99 – 1.89 (m, 4H), 1.85 – 1.78 (m, 2H), 1.65 – 1.26 (m, 48H), 0.89 (t, J = 6.0 Hz, 3H), 0.77 (d, J = 6.8 Hz, 3H).

^{13}C NMR (100 MHz, $CDCl_3$) δ (ppm) 155.7, 154.7, 152.9, 138.9, 136.5, 135.8, 132.2. 132.0, 131.7, 127.9, 126.5, 126.3, 126.1, 125.5, 125.2, 124.6, 124.6, 123.9, 123.0, 121.8, 119.7, 109.5, 109.4, 105.2,69.2, 69.1, 68.9, 63.2, 36.8, 32.9, 32.1, 30.6, 29.8, 29.8, 29.8, 29.8, 29.7, 29.7, 29.6, 29.6, 29.5, 29.5, 29.4, 29.3, 26.3, 26.2, 25.9, 22.8, 18.5, 14.3.

HRMS (ESI): calcd. for $C_{59}H_{84}O_5S_2$ [M+H] 937.5833, found 937.5812.

Motor amphiphile (MA$_{P1}$):

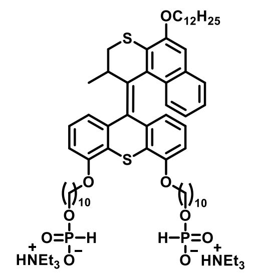

MA$_{P1}$

To a pyridine solution (3 mL) of compound **8** (110 mg, 0.117 mmol), diphenyl phosphite (192 mg, 0.819 mmol) was added and the mixture was stirred at 25 °C for 16 h. Subsequently, a solution of triethylamine/water (v/v = 1/1, 2 mL) was added and the reaction mixture was continuously stirred at 25 °C for 1 h. The solvent was evaporated at reduced pressure and the residue was subjected to column chromatography on SiO_2 (methanol (with 10% triethylamine)/dichloromethane; v/v = 1/9, R_f = 0.5) to allow for the isolation of **MA$_{P1}$** (48 mg, 0.0379 mmol, 32% yield) as a pale yellow oil.

^{1}H NMR (400 MHz, $CDCl_3$) δ (ppm) 7.59 (s, 1H), 7.42 (m, 2H), 7.26 (dd, J = 5.6, 5.6 Hz, 1H), 7.15 (d, J = 7.6 Hz, 1H), 7.02 (dd, J = 7.6, 7.6 Hz, 1H), 6.92 (s, 1H), 6.84 – 6.80 (m, 2H), 6.33 (dd, J = 8.0, 8.0 Hz, 1H), 6.25 (d, J = 8.0 Hz, 1H), 6.04 (d, J = 8.0 Hz, 2H), 4.18 – 4.10 (m, 3H), 4.07 –4.00 (m, 2H), 3.97 – 3.91 (m, 1H), 3.88 – 3.83 (m, 5H), 3.73 (dd, J = 11.2, 6.8 Hz, 1H), 3.09 – 2.99 (m, 13H), 1.97 – 1.86 (m, 4H), 1.82 – 1.75 (m, 2H), 1.66 – 1.26 (m, 66H), 0.87 (t, J = 6.4 Hz, 3H), 0.74 (d, J = 6.8 Hz, 3H).

^{13}C NMR (150 MHz, $CDCl_3$) δ (ppm) 155.6, 154.7, 152.9, 138.8, 136.4, 135.8, 132.1, 131.9, 131.7, 127.8, 126.5, 126.3, 126.1, 125.5, 125.2, 124.6, 124.5, 123.9, 122.9, 121.7, 119.6, 109.4, 109.4, 105.1, 69.2, 69.1, 68.8, 64.2, 64.2, 45.5, 36.7, 32.0, 30.9, 30.9, 30.6, 29.8, 29.8, 29.7, 29.7, 29.7, 29.6, 29.6, 29.6, 29.5, 29.5, 29.4, 29.4, 29.3, 29.3, 26.2, 26.2, 26.1, 26.0, 22.8, 18.4, 14.2, 8.6.

HRMS (ESI): calcd. for $C_{59}H_{86}O_9P_2S_2$ [M-H] 1063.5105, found 1063.5129.

Motor amphiphile (MA$_{S1}$):

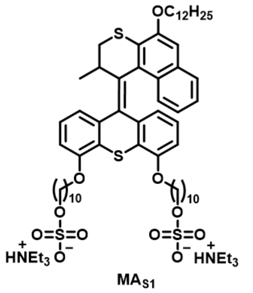

MA$_{S1}$

A mixture of compound **8** (36 mg, 0.038 mmol), sulfur trioxide pyridine complex (37 mg, 0.23 mmol) and triethylamine solution (0.1 mL) in dichloromethane (6 mL) was stirred at 25 °C for 18 h. The solvent was removed under reduced pressure and the residue was purified by column chromatography on SiO_2 (methanol (with 10% triethylamine)/dichloromethane; v/v = 1/9, R_f = 0.5) to obtain **MA$_{S1}$** (23 mg, 0.018 mmol, 47% yield) as a pale yellow oil.

^{1}H NMR (400 MHz, $CDCl_3$) δ (ppm) 9.58 (s, 2H), 7.42 (m, 2H), 7.26 (dd, J = 8.0, 8.0 Hz, 1H), 7.15 (d, J = 7.6 Hz, 1H), 7.03 (dd, J = 7.6, 7.6 Hz, 1H), 6.92 (s, 1H), 6.83 – 6.80 (m, 2H), 6.33 (dd, J = 8.0, 8.0 Hz, 1H), 6.25 (d, J = 8.0 Hz, 1H), 6.04 (d, J = 7.6 Hz, 1H), 4.17 – 4.10 (m, 3H), 4.07 – 4.01 (m, 6H), 3.97 – 3.83 (m, 2H), 3.73 (dd, J = 11.6, 6.4 Hz, 1H), 3.15– 3.06 (m, 13H), 1.97 – 1.86 (m, 4H), 1.82 – 1.75 (m, 2H), 1.70 – 1.63 (m, 4H), 1.60 – 1.26 (m, 60H), 0.87 (t, J = 6.0 Hz, 3H), 0.74 (d, J = 6.4 Hz, 3H).

^{13}C NMR (150 MHz, $CDCl_3$) δ (ppm) 155.7, 154.7, 152.9, 138.8, 136.4, 135.8, 132.1, 131.9, 131.7, 127.9, 126.5, 126.3, 126.1, 125.5, 125.1, 124.6, 124.5, 123.9, 122.9, 121.7, 119.6, 109.5, 109.4, 105.1, 69.2, 69.1, 68.8, 68.2, 54.9, 46.6, 36.7, 32.0, 30.6, 29.8, 29.7, 29.7, 29.7, 29.7, 29.6, 29.6, 29.5, 29.5, 29.5, 29.4, 29.4, 29.4, 29.3, 29.2, 26.2, 26.2, 26.1, 26.0, 22.8, 18.4, 14.2, 8.8.

HRMS (ESI): calcd. for $C_{59}H_{84}O_{11}S_4$ [M-H] 1095.4812, found 1095.4813.

4. Nile Red Fluorescence Assay.[3–5] The self-assembly properties of **MA$_{P1}$** and **MA$_{S1}$** were analyzed by incorporation of the hydrophobic solvatochromic probe Nile Red (9-diethylamino-5-benzo[α]phenoxazinone), which shows a blue shift of the emission wavelength when it is encapsulated in hydrophobic environments. Freshly prepared Nile Red ethanol solution (0.1 mM) was diluted into **MA** solutions to a final concentration of 0.25 μM, which means that the corresponding samples contain

only 0.25% ethanol, allowing to eliminate the effects of ethanol on the self-assembly behavior of $\mathbf{MA_{P1}}$ and $\mathbf{MA_{S1}}$. Subsequently, the mixture solutions containing $\mathbf{MA_{P1}}$ (or $\mathbf{MA_{S1}}$) and Nile Red solution (0.25 μM) were excited at 550 nm, and the fluorescence spectra with a wavelength range of 580–750 nm were recorded by using a JASCO FP6200 fluorometer. The blue shifts were calculated by subtracting the emission wavelength of Nile Red in Milli-Q water from the emission wavelength of the sample. Afterward, critical aggregation concentrations (CACs) of $\mathbf{MA_{P1}}$ and $\mathbf{MA_{S1}}$ were determined by plotting the obtained blue shifts values against the concentrations.

5. Cryogenic Transmission Electron Microscopy. The $\mathbf{MA_{P1}}$ solution (or $\mathbf{MA_{S1}}$ solution) was prepared by direct dissolution into double deionized water, followed by heating at 80 °C for 30 min and then cooling to room temperature to obtain a clear solution (3.9 mM). To observe the self-assembly structures by cryo-transmission electron microscopy (cryo-TEM), 2.5 μL of $\mathbf{MA_{P1}}$ solution (or $\mathbf{MA_{S1}}$ solution in identical conditions) was placed on a glow-discharged holy carbon coated grid (Quantifoil 3.5/1, QUANTIFOIL Micro Tools GmbH, Großlöbichau, Germany). After blotting, the corresponding grid was rapidly frozen in liquid ethane (Vitrobot, FEI, Eindhoven, The Netherlands) and kept in liquid nitrogen until measurement. The grids were observed with a Gatan model 626 cryo-stage in a Tecnai T20 (FEI, Eindhoven, The Netherlands) cryo-electron microscope operating at 200 keV. Cryo-TEM images were recorded under low-dose conditions on a slow-scan CCD camera.

6. Standardized Preparation of Artificial Muscle of Motor Amphiphile in Water. Artificial muscles of motor amphiphiles (**MAs**) were prepared with the standardized conditions as follows: by using a pipette, 3.5 μL of **MA** solution (55 mM) was manually drawn into an aqueous solution of $CaCl_2$ (150 mM) in a cuvette by the shear-flow method, allowing for the formation of a noodle-like artificial muscle, with a diameter of 300 ± 30 μm and a length of 8.0 ± 2.0 mm (Figure 6). The light source (Thorlabs, M365FP1, 0.7 A) was placed at a distance of 5.0 ± 0.5 mm toward the artificial muscle. The photoactuating speed of the **MA** artificial muscle was calculated from a saturated flexion angle, *i.e.*, 90°, of bending depending on a particular time. All the photoactuation experiments were performed in triplicate and recorded with a Nikon Coolpix A900 Digital Camera to obtain an averaged value of actuation speed. On the basis of the ^{1}H NMR and UV-Vis absorption studies of **MA** solutions shown in Figure 2, Figure S2, Figure 3, and Figure S4, a ratio of metastable-**MAs** : stable-**MAs** at the photostationary state can be determined as 85:15, which was comparable to our previous report.[2,5] Therefore, a similar extent of switching process of **MAs** in the artificial muscles was expected when the **MA** artificial muscles reached the saturated flexion angle of 90°.

7. Synchrotron Radiation X-ray Diffraction Analysis.[2,5,6] Through view X-ray diffraction (XRD) images of the **MA** artificial muscles were obtained using the BL45XU beamline at SPring-8 (Hyogo, Japan) equipped with an R-AXIS IV++ (Rigaku) imaging plate area detector or with a Pilatus3X 2M (Dectris) detector. The scattering vector, $q = 4\pi\sin\theta/\lambda$, and the position of incident X-ray beam on the detector were calibrated using several orders of layer reflections from silver behenate (d = 58.380 Å), where 2θ and λ refer to the scattering angle and wavelength of the X-ray beam (1.0 Å), respectively. The sample-to-detector distances for through-view XRD measurements were 2.02 m. The obtained diffraction patterns and images were integrated along the Debye-Scherrer ring to afford one-dimensional (1D) intensity data using the FIT2D software. The lattice parameters were refined using the CellCalc ver. 2.10 software.

8. Scanning Electron Microscopy and Polarized Optical Microscopy Analysis.[2,5,6] The **MA$_{P1}$** solution (or **MA$_{S1}$** solution) was prepared by heating at 80 °C for 30 min and then cooling to room temperature to obtain a clear solution (55 mM). By using a pipette, 3.5 μL of the **MA** solution was manually sheared into an aqueous solution of $CaCl_2$ (150 mM) on a sapphire substrate to obtain a noodle-like **MA** artificial muscle with an arbitrary length. After the removal of $CaCl_2$ solution, the **MA** artificial muscle was washed with deionized water (three times), and the resulting **MA** artificial muscle was used directly for polarized optical microscopy (POM, Nikon model Eclipse LV100POL) and XRD experiments. For scanning electron microscopy (SEM), the **MA** artificial muscle was dried in air for 48 h and subsequently observed under a Hitachi S-5500 Field Emission SEM (FE-SEM).

9. Cell Culture.[7,8] Human bone marrow-derived mesenchymal stem cells (**hBM-MSCs**) were obtained from Lonza. A growth medium consisted of Alpha modified Eagle medium (Gibco), 10% (v/v) fetal bovine serum (Gibco), 0.1% (v/v) ascorbic acid 2-phosphate (Sigma), and 2% penicillin/streptomycin (Gibco). **hBM-MSCs** were incubated in T75 culture flasks with the conditions of 37 °C and 5% CO_2. The growth medium was changed every 3 d and the **hBM-MSCs** were detached with trypsin for 3 – 5 min at 37 °C and harvested at about 80 – 90% confluence. The corresponding **hBM-MSCs** were used for cell culture experiments.

Freshly prepared artificial muscles of **MA$_{C1}$** and **MA$_{P1}$** were placed in 24-well plates, which were washed with PBS (0.5 mL) 3 times and subsequently washed by growth medium (0.5 mL) 3 times. Afterward, the **hBM-MSCs** with particular densities were evenly seeded into the growth medium containing **MA** artificial muscles. The samples were incubated in a 5% CO_2 humidified atmosphere at 37 °C. The growth medium was changed every 3 d.

10. Cytotoxicity and Cell Viability Analysis. The *vitro* cytotoxicity of **MA** artificial muscles was tested by using a direct contact method between **hBM-MSCs** and **MA** artificial muscles via a live/dead staining assay.[9] The **hBM-MSCs** with a density of 20,000 cells/well were evenly seeded into 24-well plates containing growth medium and **MA** artificial muscles, and incubated in a 5% CO_2 humidified atmosphere at 37 °C. After incubation for 24 h, the cytotoxicity of **hBM-MSCs** was analyzed by the live/dead assay by using calcein-AM (2 μL, Molecular Probes, Invitrogen Detection Technologies) and ethidium homodimer-1 (4 μL, Molecular Probes, Invitrogen Detection Technologies) in PBS to stain the **hBM-MSCs** for 30 min.[7] After staining, the **MA** artificial muscles were moved out from the 24-well plates to a well plate (diameter: 60 mm, containing PBS) for being observed under a Leica fluorescence microscopy equipped with a 10× NA 0.30 objective and a Leica confocal laser scanning microscopy (CLSM) equipped with a 40× NA 0.80 objective.

Additionally, a deeper insight into the condition of **hBM-MSCs** cultured on the surface of the **MA** artificial muscles with prolonged incubation time is performed by F-actin and nuclei staining. The **hBM-MSCs** with a density of 2,500 cells/well were evenly seeded into 24-well plates containing growth medium and **MA** artificial muscles and incubated in a 5% CO_2 humidified atmosphere at 37 °C for 1, 3, and 5 d. After incubation, the **hBM-MSCs** were fixed with 3.7% paraformaldehyde in phosphate-buffered saline (PBS) for 20 min at room temperature and then washed by PBS for three times. Subsequently, the cell membrane was permeabilized with a 0.5% TritonX-100 solution for 3 min, followed by washing three times with PBS. The obtained **hBM-MSCs** were stained with 4′,6-diamidino-2-phenylindole (DAPI) and tetramethylrhodamine isothiocyanate(TRITC)–phalloidin for the cell nuclei and F-actin, respectively, which were observed under the Leica confocal laser scanning

microscopy equipped with a 40× NA 0.80 objective.[7] The cell nuclei stained by DAPI were not shown because of the strong background fluorescence of the **MA** artificial muscles. The area of cell F-actin and the surface area of the **MA** artificial muscles were determined by the software of ImageJ. A spread cell shape with well-defined actin stress fibers of **hBM-MSCs** is commonly quantified as being in a viable state. Due to the overlapping fluorescence between the strong background of **MA** artificial muscles and the cell nuclei stained by DAPI, the change of cell F-actin area with prolonged incubation time, instead of the number of cell nuclei, was used to indicate the cell proliferation.

11. Post-Photoactuation Experiment of Artificial Muscles with Adhered hBM-MSCs. The **hBM-MSCs** with a density of 20,000 cells/well were evenly seeded into 24-well plates containing growth medium and **MA** artificial muscles, and incubated in a 5% CO_2 humidified atmosphere at 37 °C for 6 h. After incubation, **hBM-MSCs** were stained by using calcein-AM (2 μL) and ethidium homodimer-1 (4 μL) in PBS for 30 min. Subsequently, the **MA** artificial muscles were moved out from the 24-well plates to a well plate (diameter: 60 mm, containing PBS) for observation under the Leica fluorescence microscopy equipped with a 10× NA 0.30 objective. The **MA** artificial muscles were irradiated by using a Thorlabs LED light (M365L2-C1, 120 mW). The photoactuation process upon irradiation was simultaneously recorded by fluorescence microscopy with cell visualization at a micro-length scale and by an iPhone camera at a macroscopic scale, as shown in Figure S1.

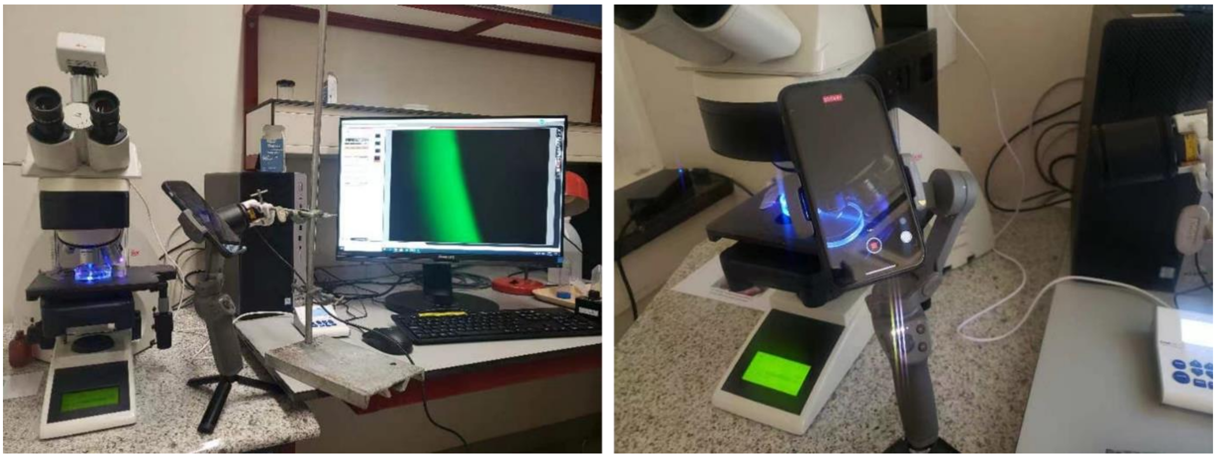

Figure S1. Photographs of instruments for photoactuation experiments of artificial muscles with adhered **hBM-MSCs**.

12. Description of Supplementary Movies.

Movie 1: Three-dimensional visualization of **MA** artificial muscles with adhered **hBM-MSCs** by using confocal laser scanning microscopy.

Movie 2: Post-Photoactuation of **MA**$_{\mathrm{C1}}$ artificial muscle with adhered **hBM-MSCs**.

Movie 3: Post-Photoactuation of **MA**$_{\mathrm{P1}}$ artificial muscle with adhered **hBM-MSCs**.

^{1}H NMR Spectra of Photoisomerization of MAs

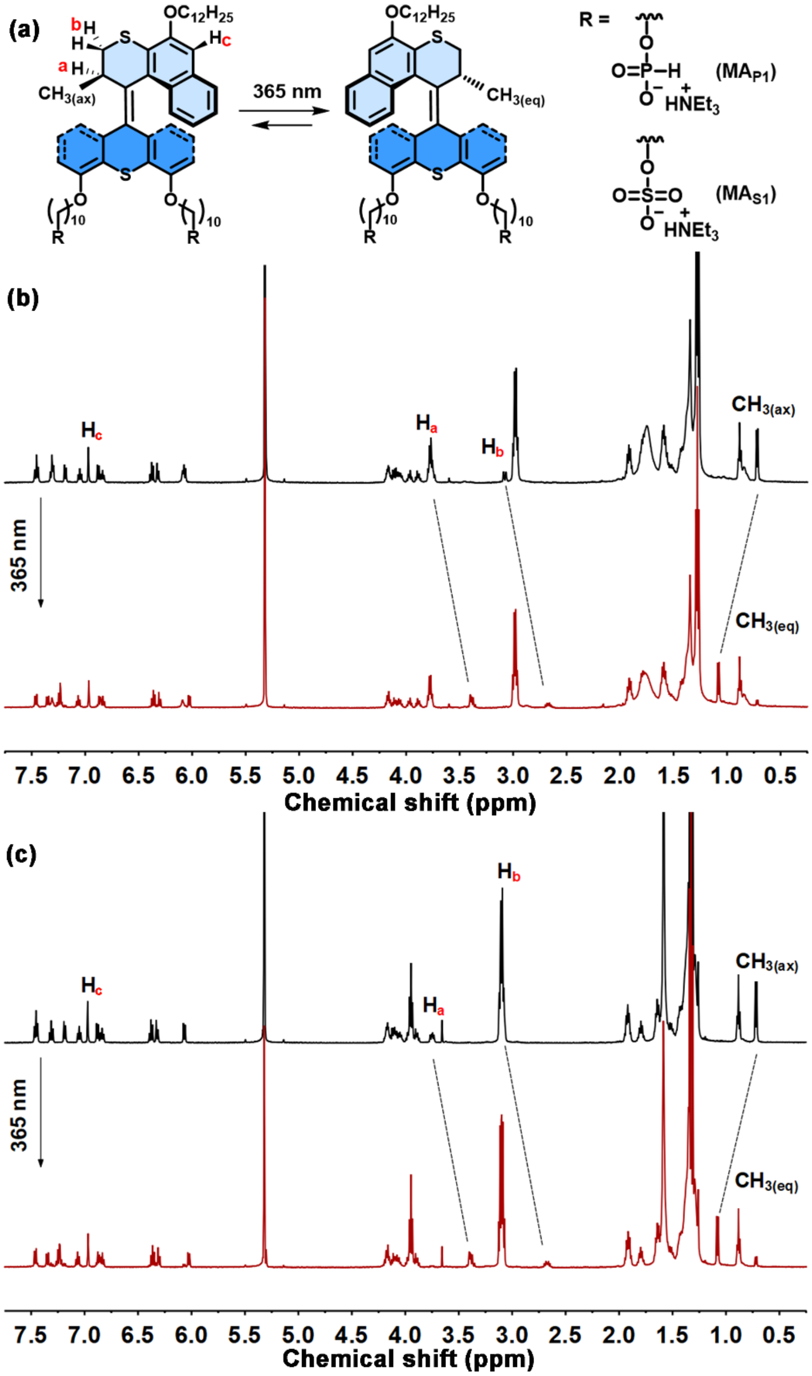

Figure S2. (a) Schematic representation of photoisomerization of **MAs**. ^{1}H NMR spectra (CD_2Cl_2, 25 °C, 500 MHz) of (b) **MA$_{P1}$** and (c) **MA$_{S1}$** in a stable state (black) and a photostationary state mixture (red) isomers after irradiation.

Table S1. Integration values of the selective protons in the ^{1}H NMR spectra (CD_2Cl_2, 25 °C, 500 MHz) of **MAs** at photostationary state after irradiation.

Samples	H_c	$CH_{3(ax)}$ (Stable isomers)	$CH_{3(eq)}$ (Metastable isomers)
**MA$_{P1}$**	1	0.47	2.61
**MA$_{S1}$**	1	0.46	2.54

Kinetic Studies of Thermal Helix Inversion Step of MA$_{P1}$ and MA$_{S1}$ in Acetonitrile

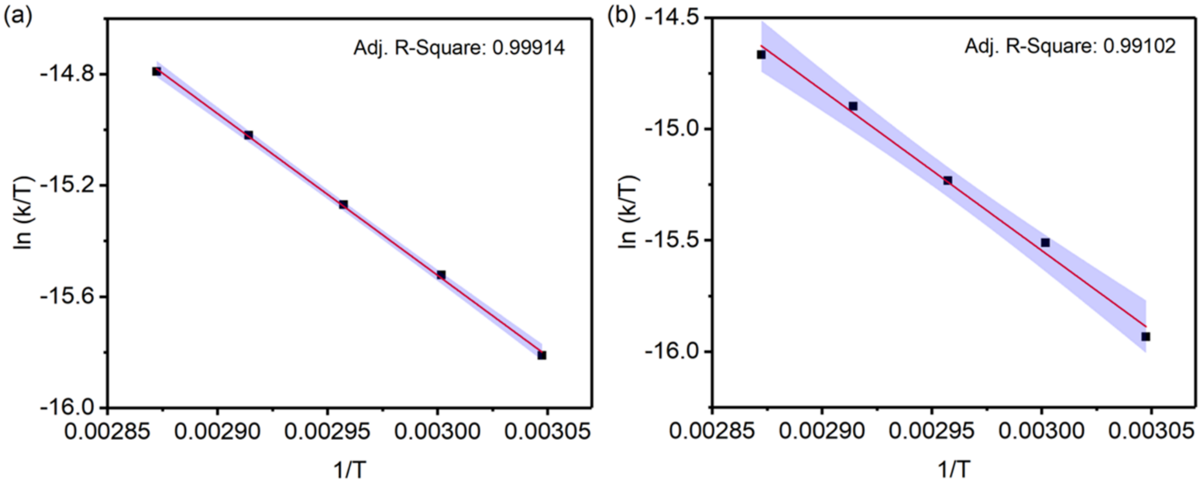

Figure S3. Kinetic studies of the thermal helix inversion step from metastable **MA** to stable **MA** of (a) **MA$_{P1}$** and (b) **MA$_{S1}$** in CH_3CN solutions at five temperatures (55 °C, 60 °C, 65 °C, 70 °C, and 75 °C) by UV-vis absorption spectral changes at 342 nm. Purple bands indicate 95% confidence interval.

The rate constants *k* of the first-order decay at different temperatures were obtained using the equation $A/A_0 = e^{-kt}$. Activation parameters were obtained by fitting to the linearized form of the Eyring equation using origin software. Analysis of these data using equation ($\Delta^{\ddagger}G = RT$ [ln (k_B/h) - ln (k/T)], provides the Gibbs energy of activation ($\Delta^{\ddagger}G$). The half-lives of metastable **MA$_{P1}$** and **MA$_{S1}$** at 25 °C (298.15 K) were obtained by a linear fitting of ln *k*/T and 1/T. The activation parameters and half-life of metastable **MA$_{P1}$** and **MA$_{S1}$** in acetonitrile are presented in Table S2.

Table S2. Activation parameters and half-lives of metastable **MA$_{P1}$** and metastable **MA$_{S1}$**.

Samples	$t_{1/2}$ at 298.15 K (h)	$\Delta^{\ddagger}G$ at 298.15 K (kJ/mol)	$\Delta^{\ddagger}H$ (kJ/mol)	$\Delta^{\ddagger}S$ (J/K/mol)
**MA$_{P1}$**	~27.9	102.5±0.9	48.4±0.7	-181.5±2.1
**MA$_{S1}$**	~46.7	103.8±3.8	59.9±2.8	-147.2±8.4

UV-vis Absorption Spectra of Photoisomerization of MAs in Water

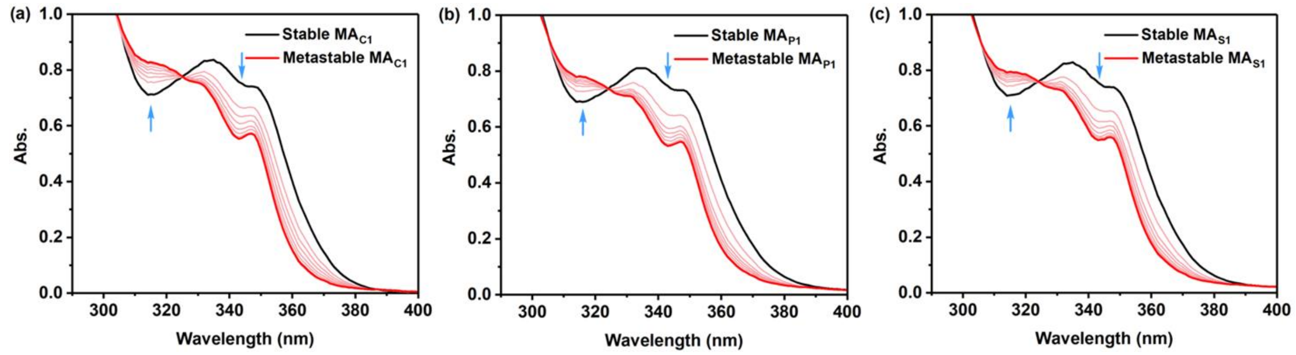

Figure S4. UV-vis absorption spectra of (a) **MA_{C1}**, (b) **MA_{P1}**, and (c) **MA_{S1}** in water before 365 nm light irradiation (black), upon irradiation from 1 min to 3 min (pink), and after irradiation to PSS (red).

Nile Red Fluorescence Assay for Determining Critical Aggregation Concentration

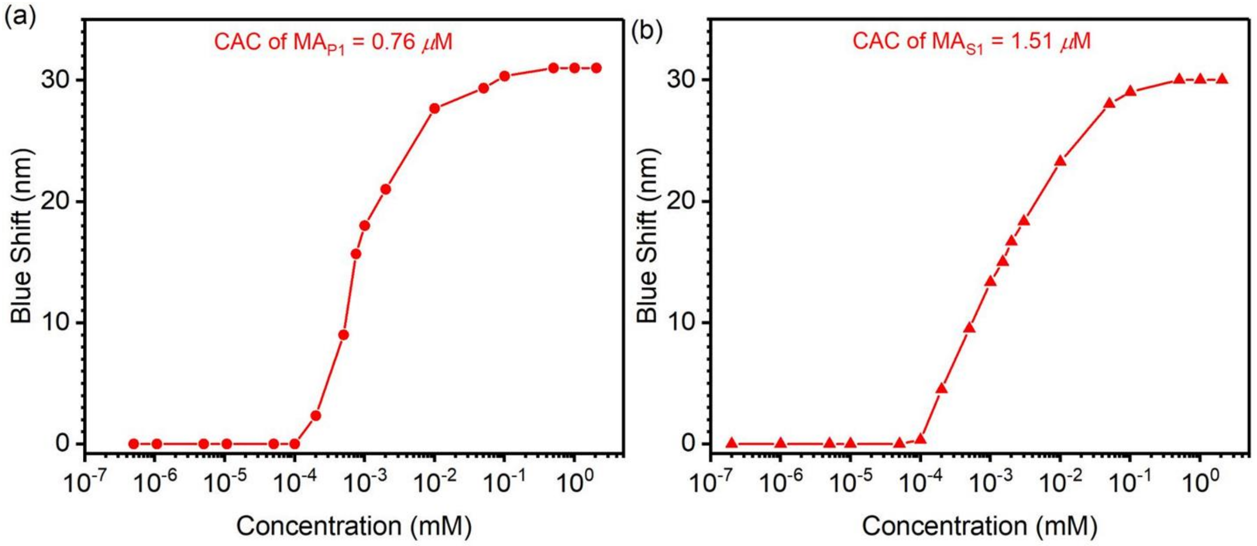

Figure S5. Nile Red fluorescence assay for the determination of critical aggregation concentration (CAC) of $\mathbf{MA_{P1}}$ and (b) $\mathbf{MA_{S1}}$.

Structural Analysis of MA$_{P1}$ Artificial Muscle by Optical and Polarized Optical Microscopy

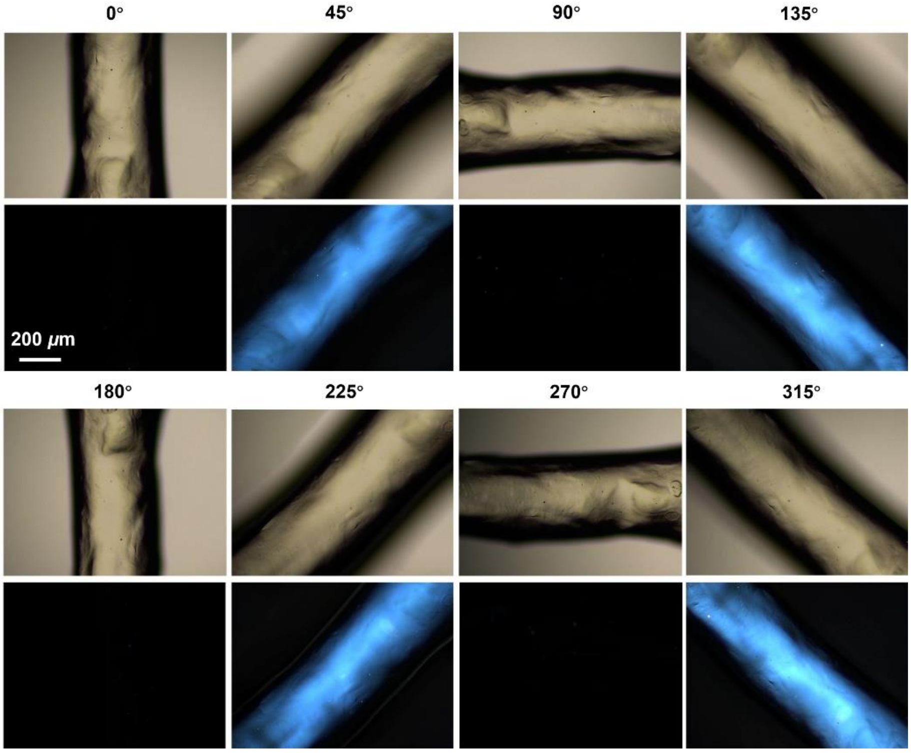

Figure S6. The OM (top) and POM (bottom) images of a noodle-like artificial muscle of **MA$_{P1}$** under crossed polarizers. The POM images of **MA$_{P1}$** artificial muscle were tilted at 0°, 45°, 90°, 135°, 180°, 225°, 270°, and 315° relative to the transmission axis of the analyzer. Scale bar applied for all panels.

Fluorescence Microscopy Images for Cytotoxicity

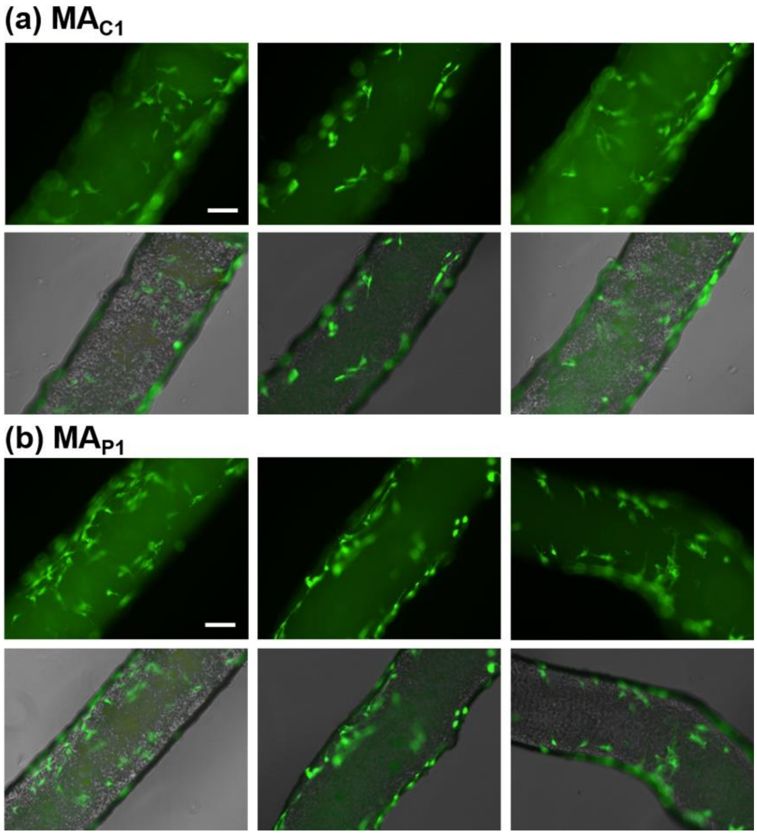

Figure S7. Cytotoxicity of artificial muscles of (a) **MA$_{C1}$** and (b) **MA$_{P1}$** for **hBM-MSCs** after 24 h incubation, determined by a live/dead staining assay in triplicate. The images were taken by fluorescence microscopy, which shows the fluorescence images (top) and the merge images combined with the bright field image (bottom). Scale bar: 100 μm, applied for all panels.

Post-Photoactuation of MA_{P1} Artificial Muscle with adhered hBM-MSCs

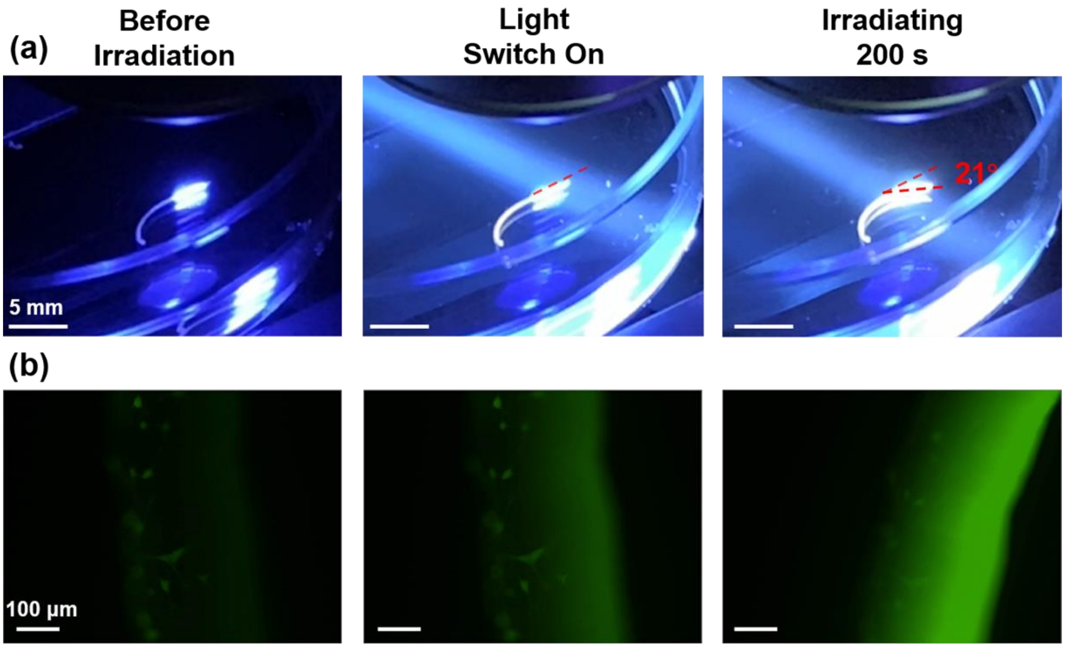

Figure S8. Snapshots showing (a) macroscopic photoactuation (scale bar: 5 mm) of an **MA_{P1}** artificial muscle with adhered **hBM-MSCs** on the surface and (b) *in situ* observation of actuation movement accompanied with cell visualization under fluorescence microscopy upon 365 nm light irradiation for 200 s (scale bar: 100 μm).

Analytical Data

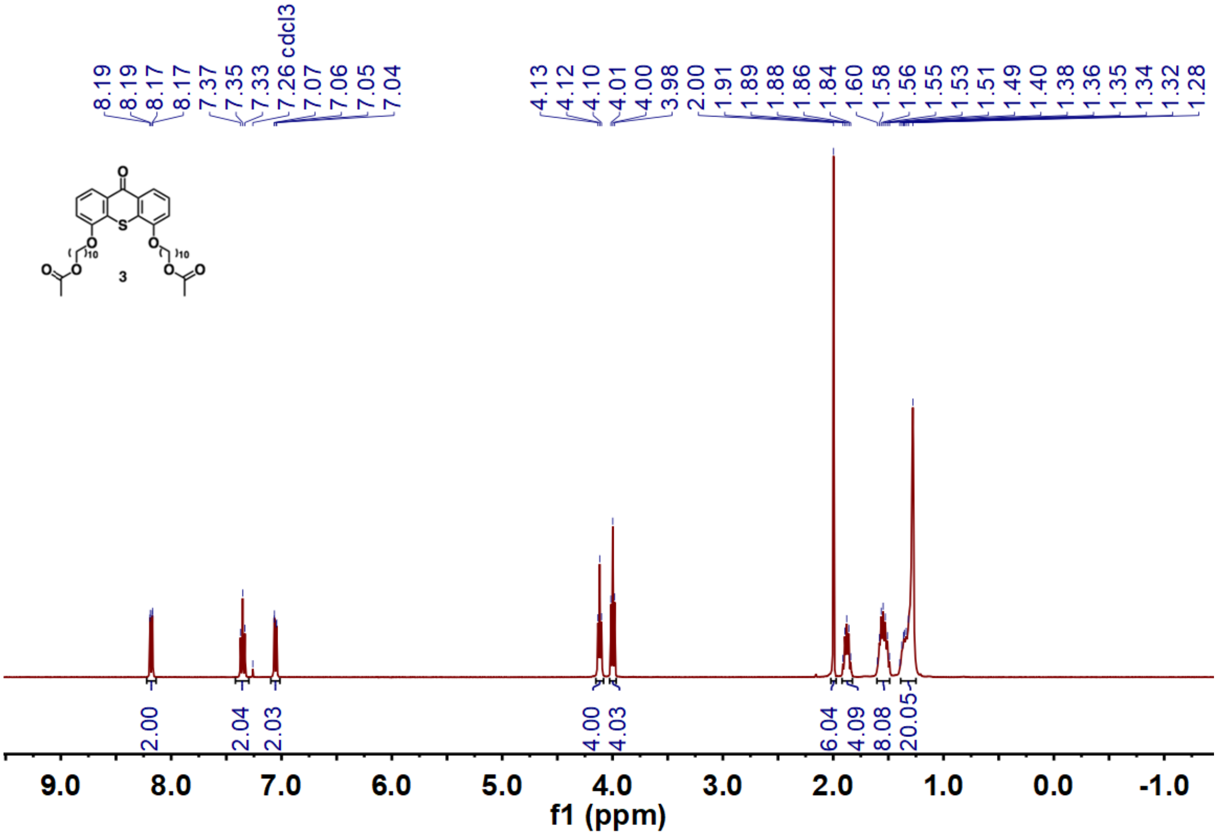

Figure S9. ^{1}H NMR spectrum (400 MHz) of **compound 3** in $CDCl_3$ at 25 °C.

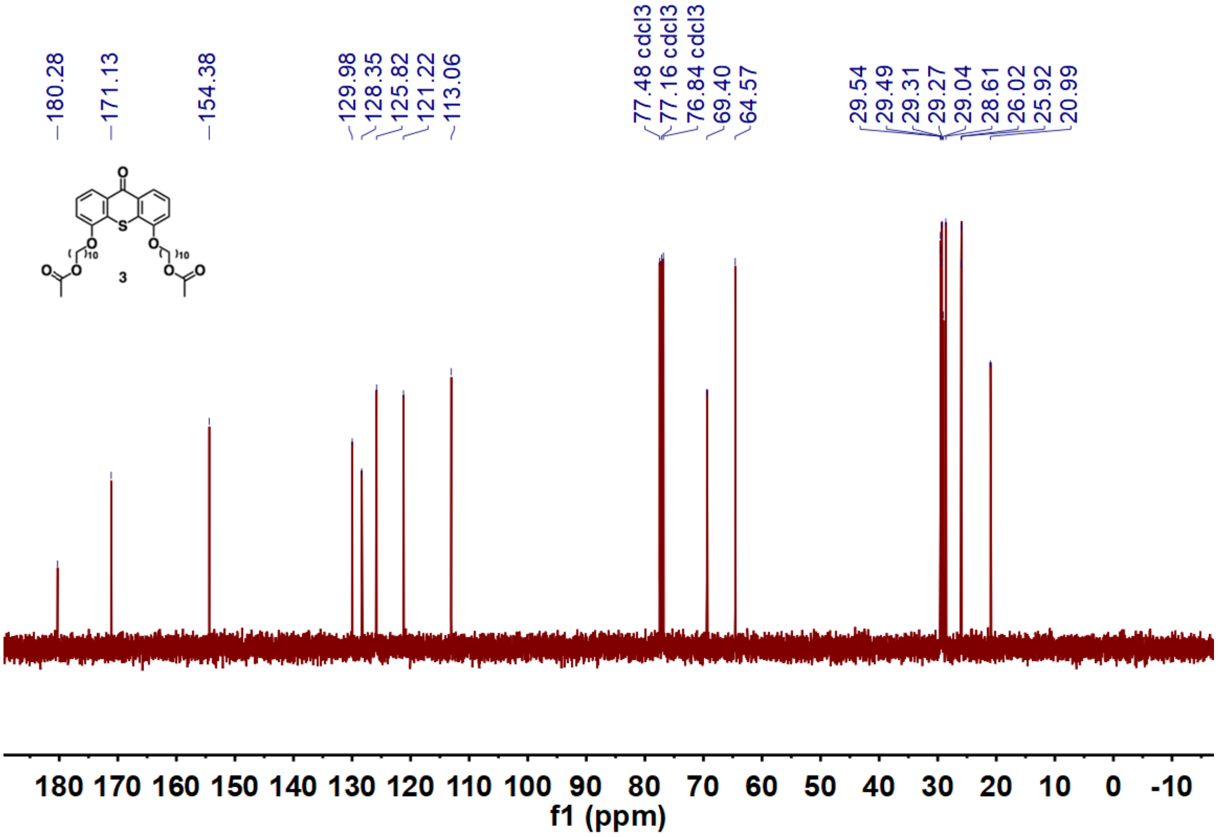

Figure S10. ^{13}C NMR spectrum (100 MHz) of **compound 3** in $CDCl_3$ at 25 °C.

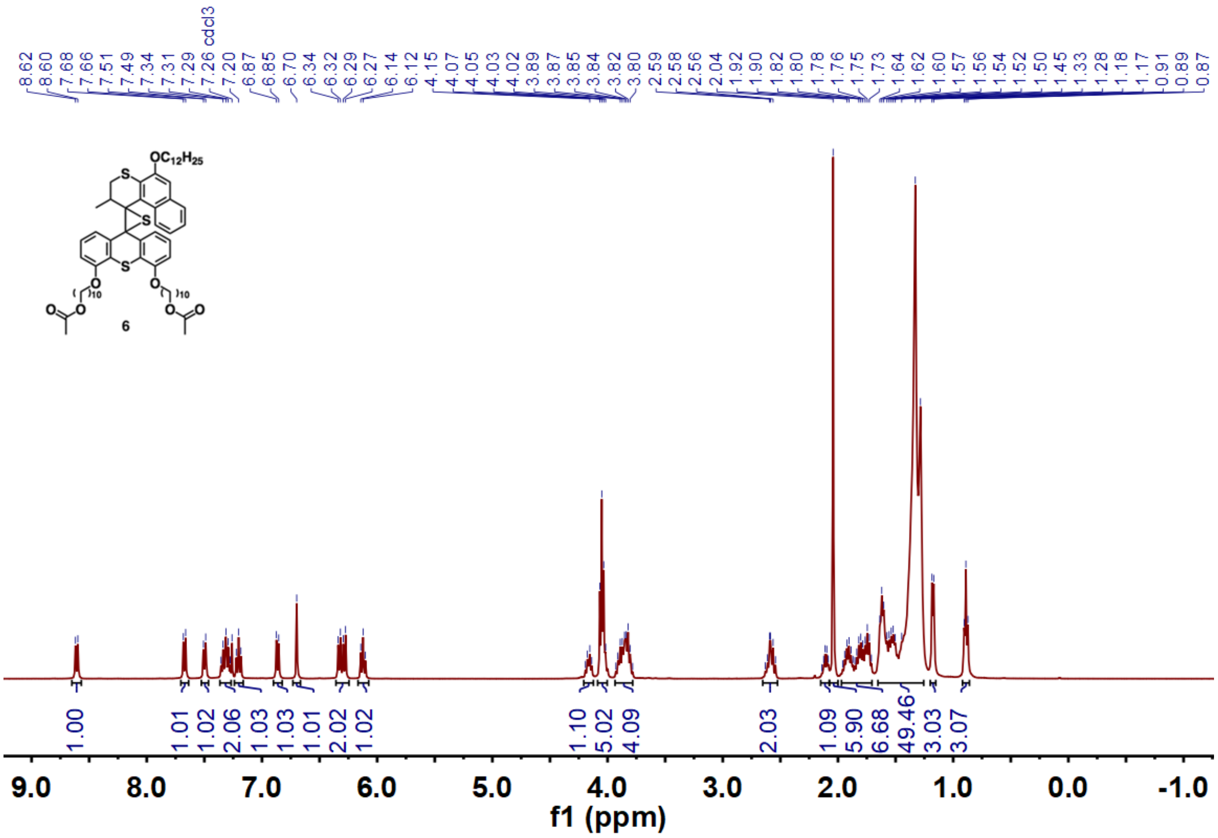

Figure S11. ^{1}H NMR spectrum (400 MHz) of **compound 6** in $CDCl_3$ at 25 °C.

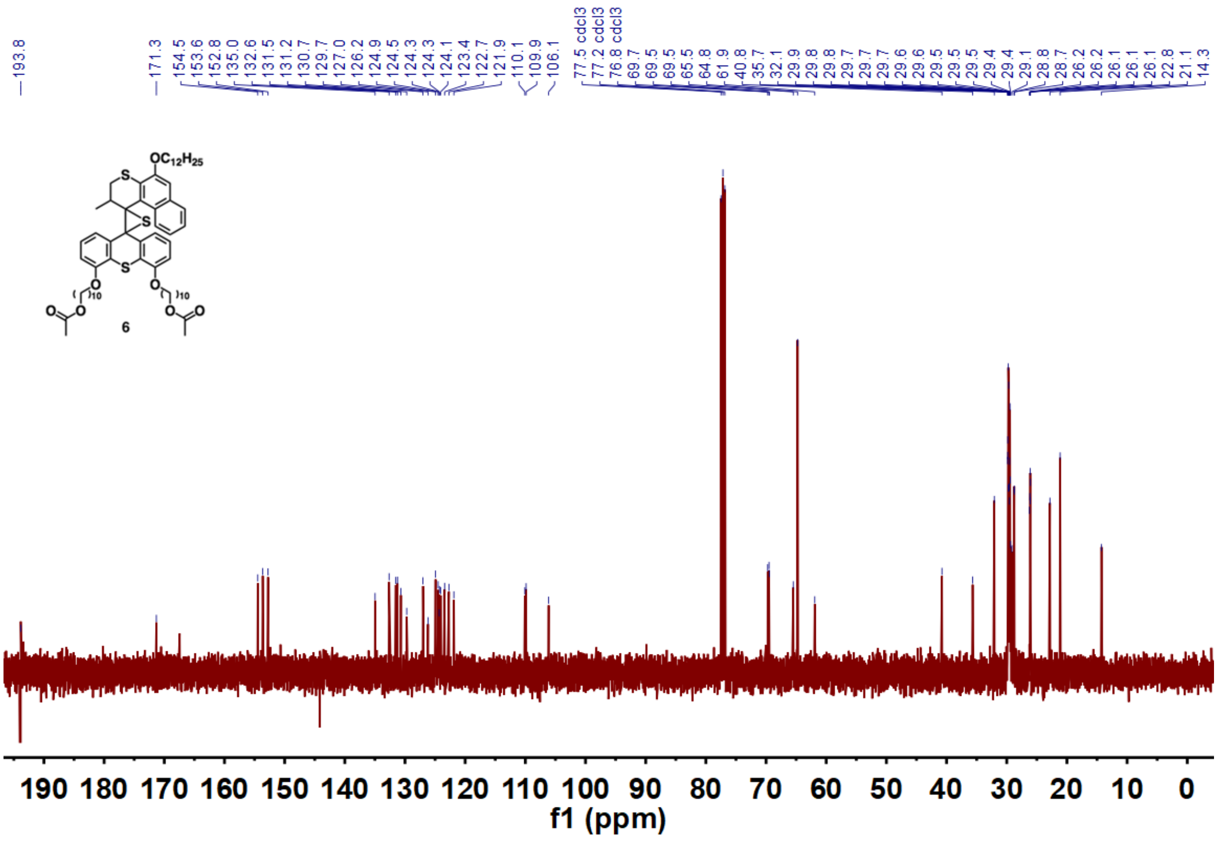

Figure S12. ^{13}C NMR spectrum (100 MHz) of **compound 6** in $CDCl_3$ at 25 °C.

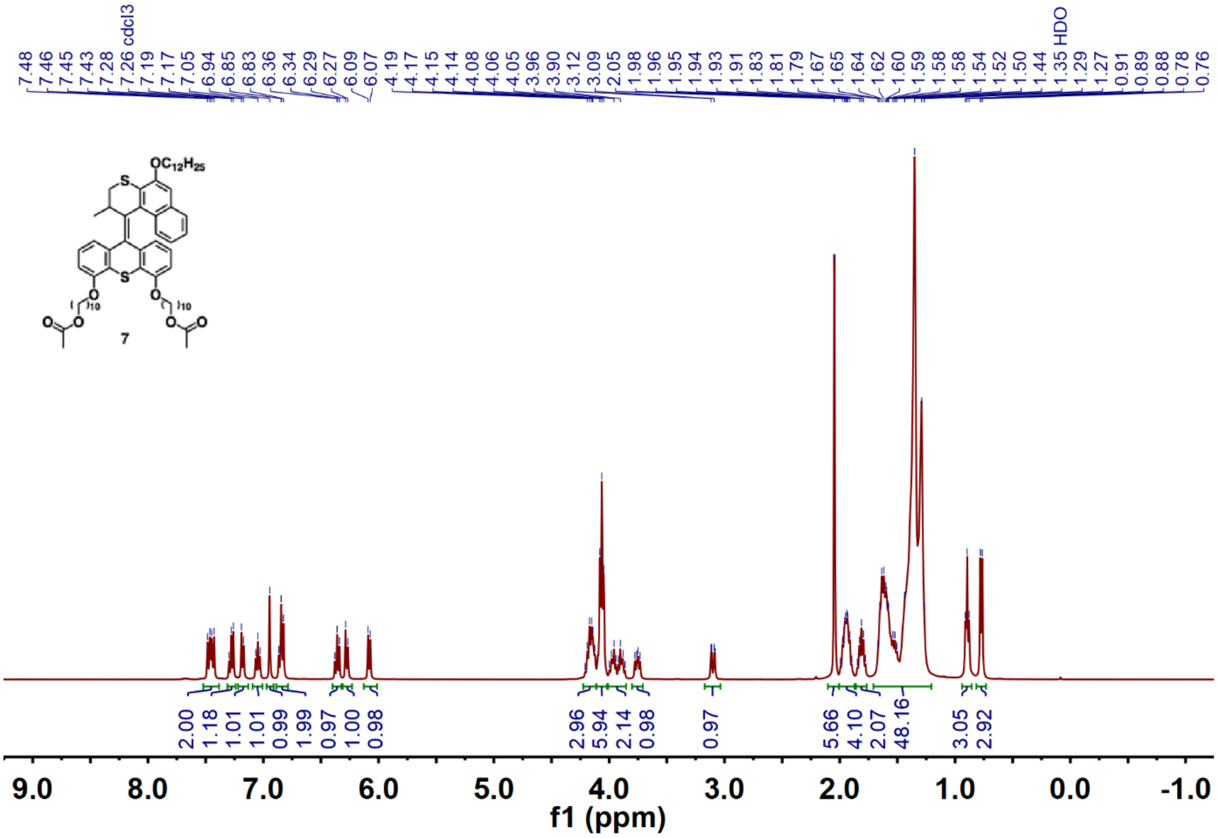

Figure S13. ^{1}H NMR spectrum (400 MHz) of **compound 7** in $CDCl_3$ at 25 °C.

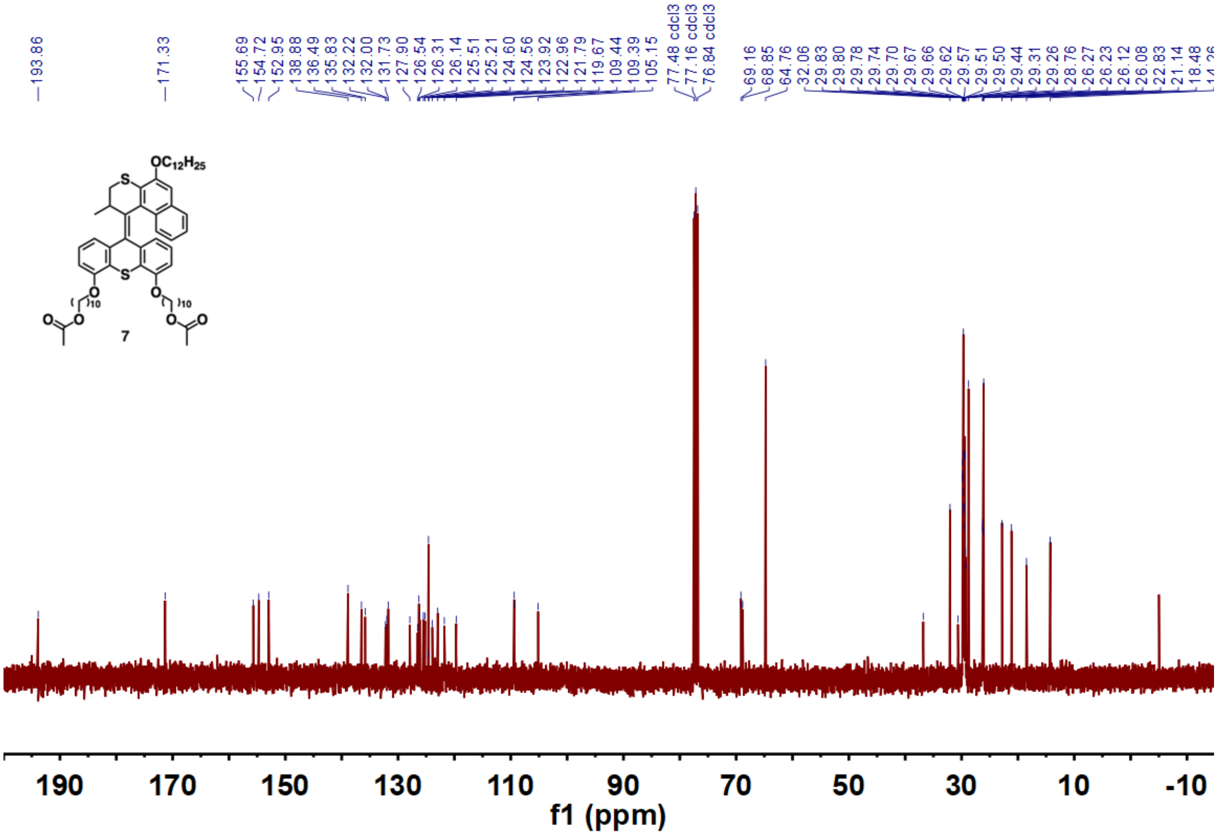

Figure S14. ^{13}C NMR spectrum (100 MHz) of **compound 7** in $CDCl_3$ at 25 °C.

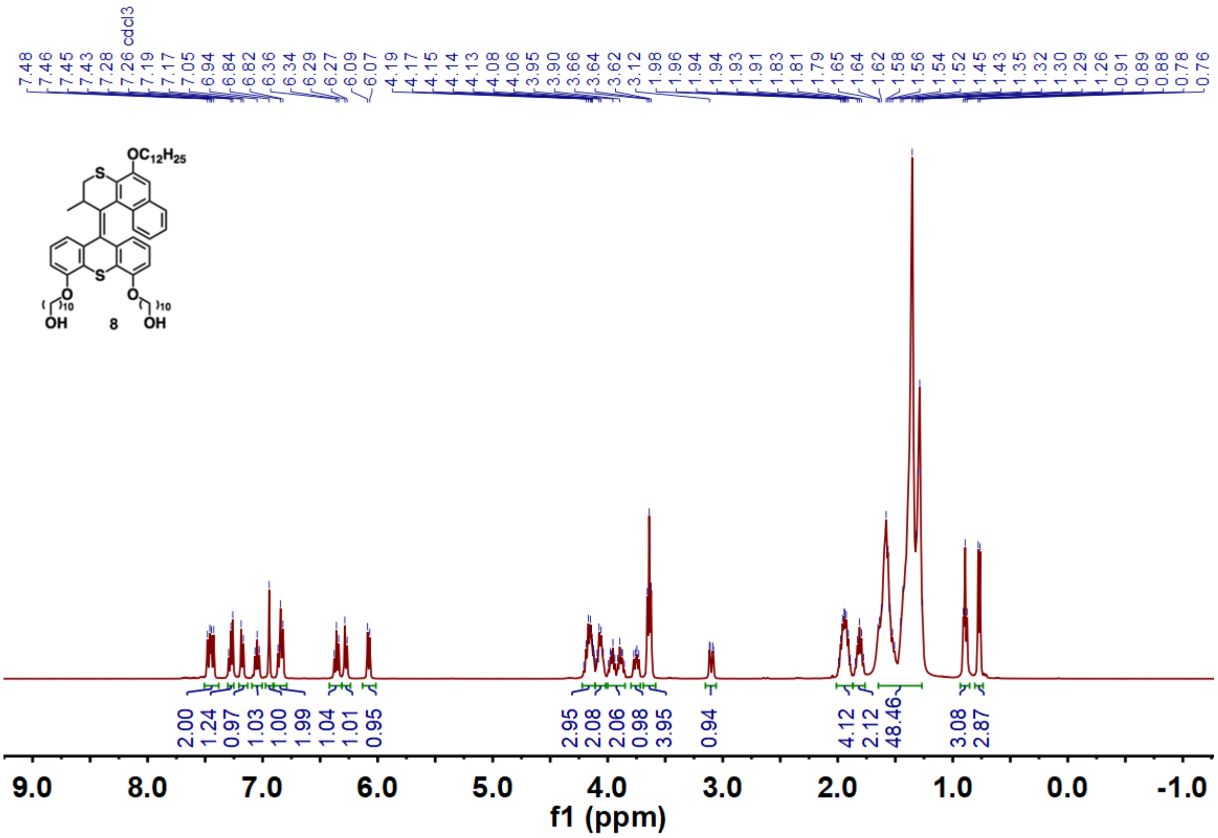

Figure S15. ^{1}H NMR spectrum (400 MHz) of **compound 8** in $CDCl_3$ at 25 °C.

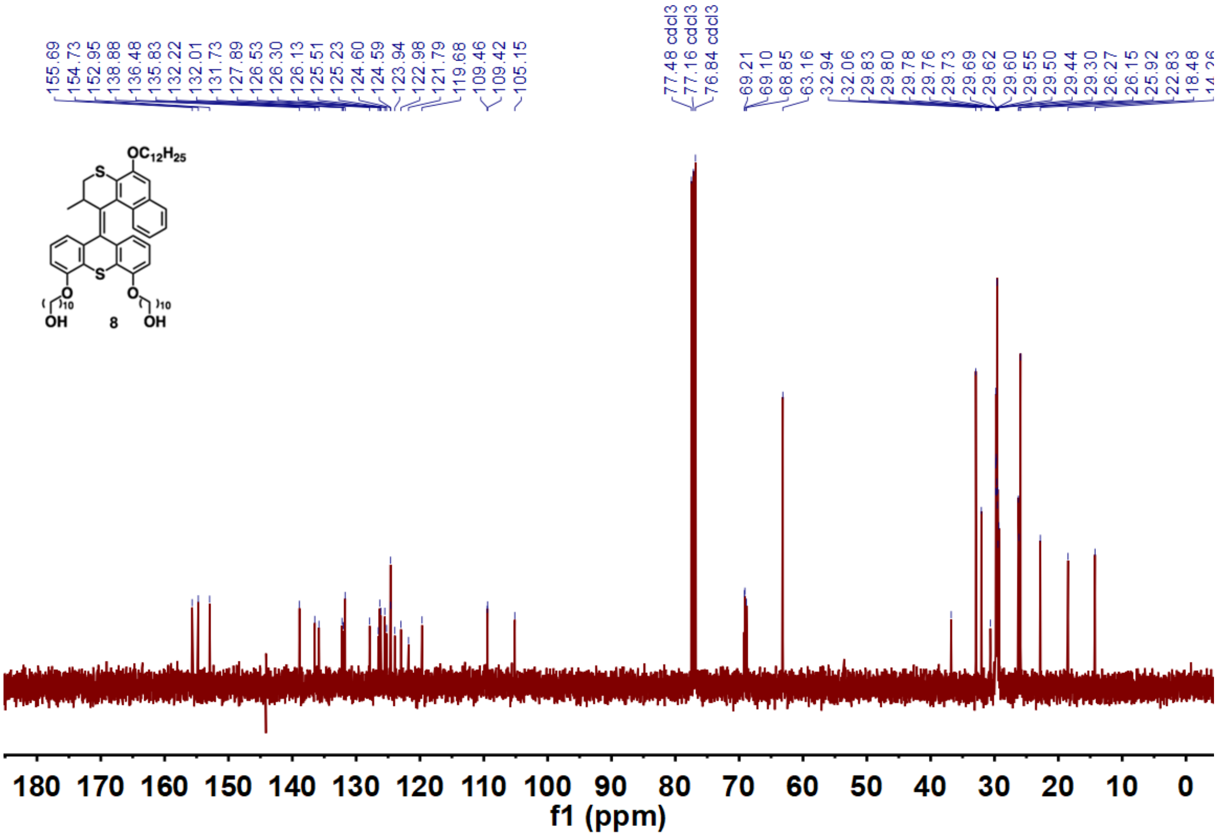

Figure S16. ^{13}C NMR spectrum (100 MHz) of **compound 8** in $CDCl_3$ at 25 °C.

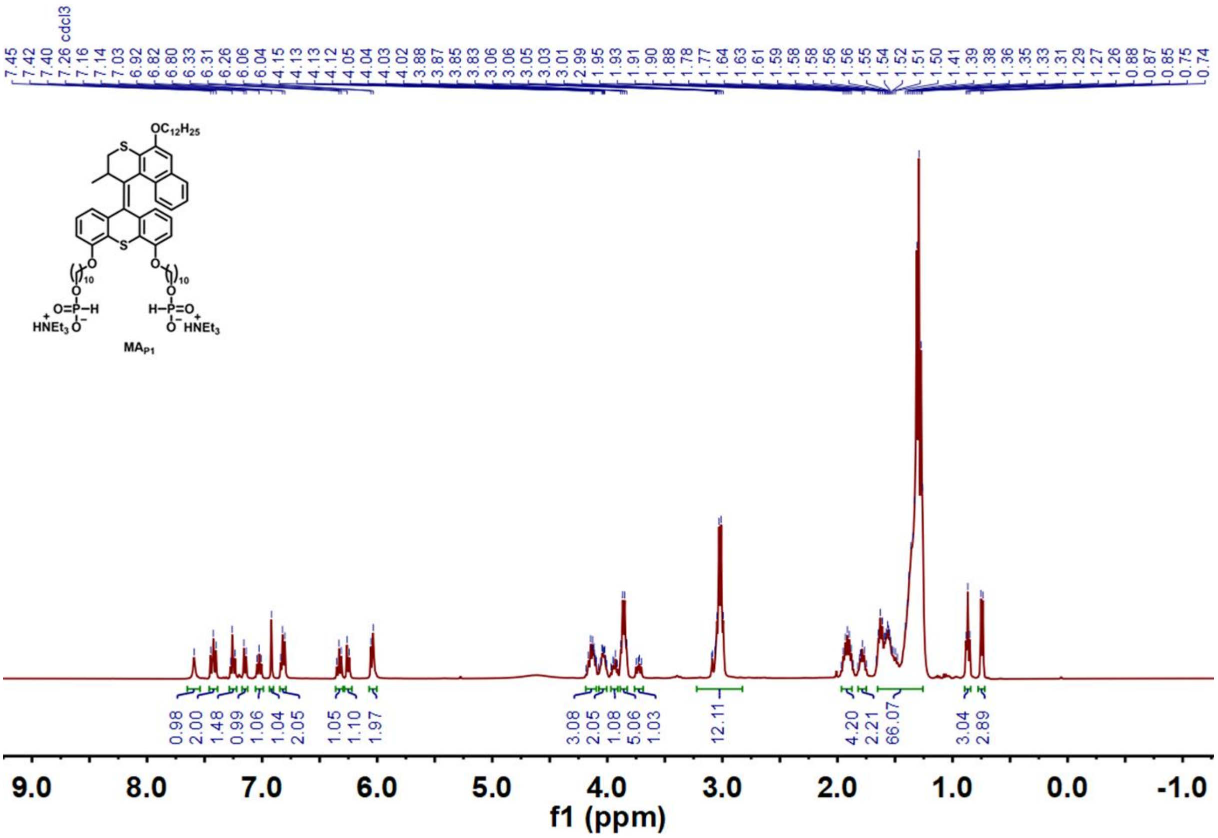

Figure S17. ^{1}H NMR spectrum (400 MHz) of **MA$_{P1}$** in $CDCl_3$ at 25 °C.

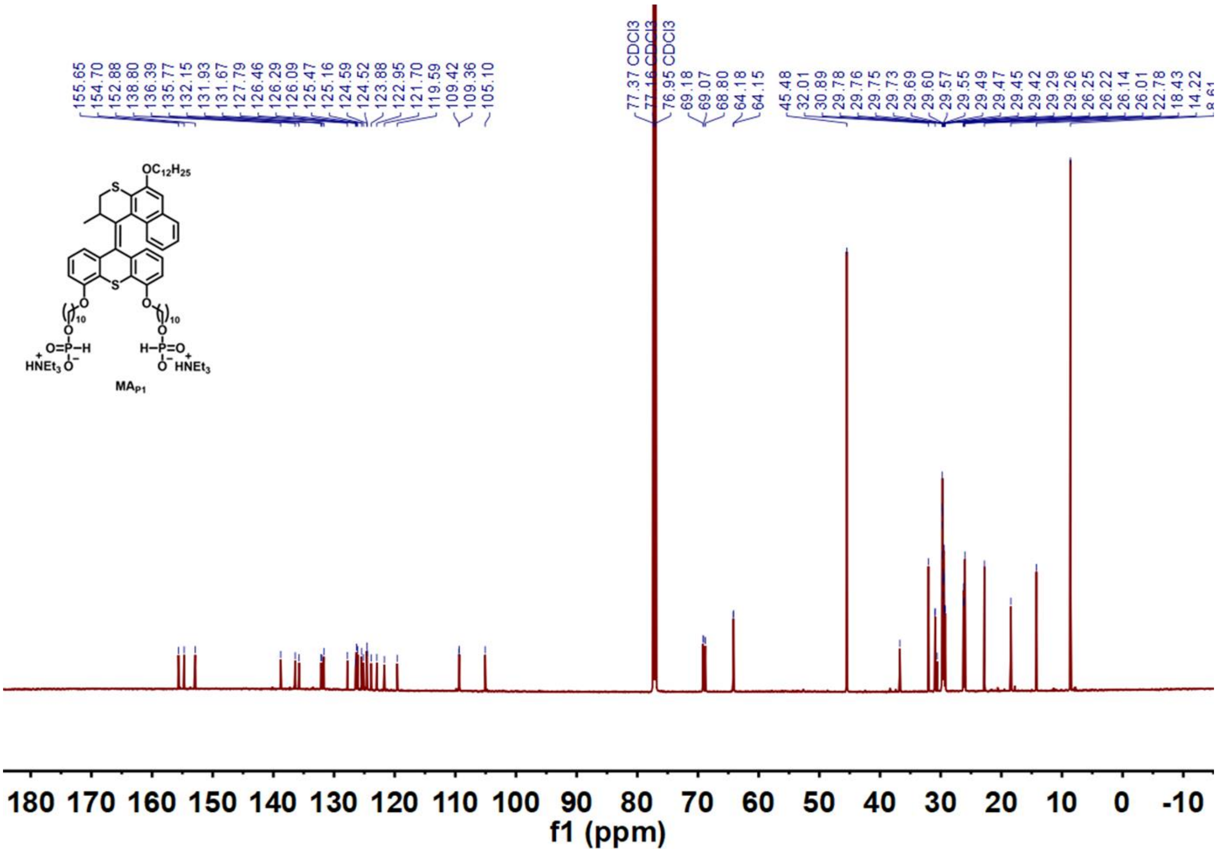

Figure S18. ^{1}C NMR spectrum (150 MHz) of **MA$_{P1}$** in $CDCl_3$ at 25 °C.

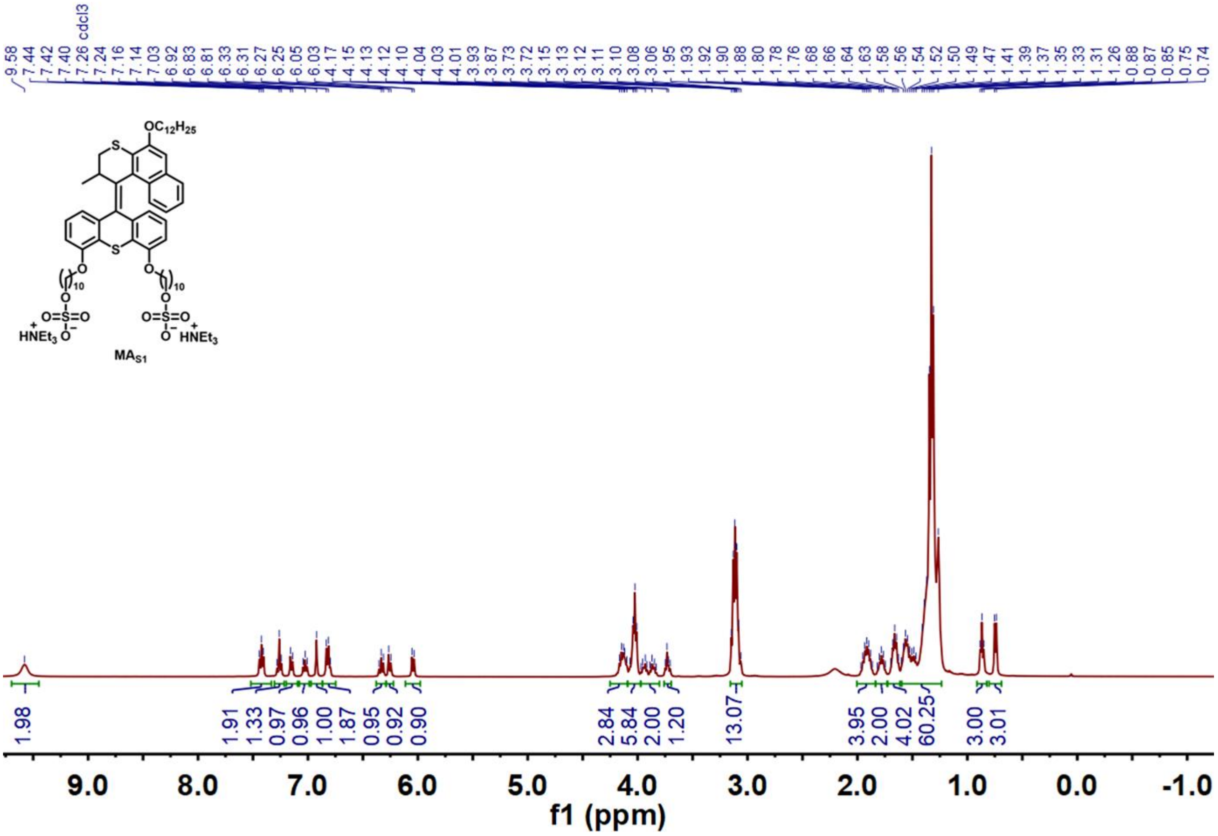

Figure S19. ^{1}H NMR spectrum (400 MHz) of $\mathbf{MA_{S1}}$ in $CDCl_3$ at 25 °C.

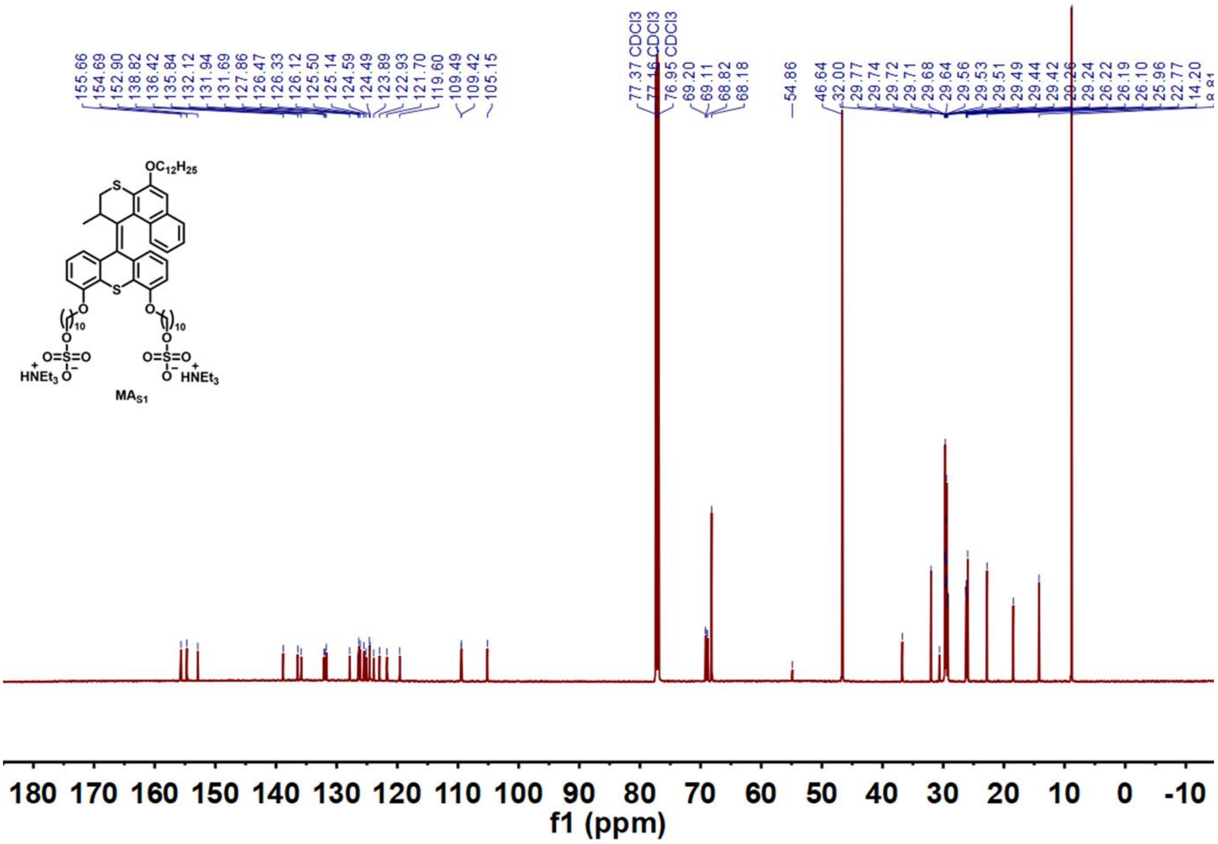

Figure S20. ^{1}C NMR spectrum (150 MHz) of $\mathbf{MA_{S1}}$ in $CDCl_3$ at 25 °C.

References

(1) van Dijken, D. J.; Chen, J.; Stuart, M. C. A.; Hou, L.; Feringa, B. L. Amphiphilic Molecular Motors for Responsive Aggregation in Water. *J. Am. Chem. Soc.* **2016**, *138*, 660–669.

(2) Chen, J.; Leung, F. K. C.; Stuart, M. C. A.; Kajitani, T.; Fukushima, T.; van der Giessen, E.; Feringa, B. L. Artificial Muscle-like Function from Hierarchical Supramolecular Assembly of Photoresponsive Molecular Motors. *Nat. Chem.* **2018**, *10*, 132–138.

(3) Stuart, M. C. A.; van de Pas, J. C.; Engberts, J. B. F. N. The Use of Nile Red to Monitor the Aggregation Behavior in Ternary Surfactant-Water-Organic Solvent Systems. *J. Phys. Org. Chem.* **2005**, *18*, 929–934.

(4) Tantakitti, F.; Boekhoven, J.; Wang, X.; Kazantsev, R. V.; Yu, T.; Li, J.; Zhuang, E.; Zandi, R.; Ortony, J. H.; Newcomb, C. J.; Palmer, L. C.; Shekhawat, G. S.; Olvera de la Cruz, M.; Schatz, G. C.; Stupp, S. I. Energy Landscapes and Functions of Supramolecular Systems. *Nat. Mater.* **2016**, *15*, 469–476.

(5) Leung, F. K. C.; van den Enk, T.; Kajitani, T.; Chen, J.; Stuart, M. C. A.; Kuipers, J.; Fukushima, T.; Feringa, B. L. Supramolecular Packing and Macroscopic Alignment Controls Actuation Speed in Macroscopic Strings of Molecular Motor Amphiphiles. *J. Am. Chem. Soc.* **2018**, *140*, 17724–17733.

(6) Leung, F. K. C.; Kajitani, T.; Stuart, M. C. A.; Fukushima, T.; Feringa, B. L. Dual-Controlled Macroscopic Motions in a Supramolecular Hierarchical Assembly of Motor Amphiphiles. *Angew. Chem. Int. Ed.* **2019**, *58*, 10985–10989.

(7) Zhou, Q.; Chen, J.; Luan, Y.; Vainikka, P. A.; Thallmair, S.; Marrink, S. J.; Feringa, B. L.; van Rijn, P. Unidirectional Rotating Molecular Motors Dynamically Interact with Adsorbed Proteins to Direct the Fate of Mesenchymal Stem Cells. *Sci. Adv.* **2020**, *6*, 2756.

(8) Yang, L.; Ge, L.; Zhou, Q.; Jurczak, K. M.; van Rijn, P. Decoupling the Amplitude and Wavelength of Anisotropic Topography and the Influence on Osteogenic Differentiation of Mesenchymal Stem Cells Using a High-Throughput Screening Approach. *ACS Appl. Bio Mater.* **2020**, *3*, 3690–3697.

(9) Peng, X.; Xia, X.; Xu, X.; Yang, X.; Yang, B.; Zhao, P.; Yuan, W.; Chiu, P. W. Y.; Bian, L. Ultrafast Self-Gelling Powder Mediates Robust Wet Adhesion to Promote Healing of Gastrointestinal Perforations. *Sci. Adv.* **2021**, *7*, 8739.
